# Expression of CD38 on resting peripheral iNKT cells defines an immature subpopulation with distinct functionality in humans

**DOI:** 10.1111/imcb.70074

**Published:** 2025-12-26

**Authors:** Christopher Menne, Naeimeh Tavakolinia, Louis Perriman, Wiebke Moskorz, Christine Cosmovici, Andreas Walker, Lara Olejnik, Katharina Raba, Mei RM Du, Fernando J Rossello, Igor E Konstantinov, Stuart P Berzins, Daniel G Pellicci, Jörg Timm

**Affiliations:** ^1^ Institute of Virology Heinrich Heine University, Medical Faculty Düsseldorf Germany; ^2^ Murdoch Children's Research Institute The Royal Children's Hospital Melbourne VIC Australia; ^3^ The Fiona Elsey Cancer Research Institute Ballarat VIC Australia; ^4^ Federation University Ballarat VIC Australia; ^5^ Institute for Transplantation Diagnostics and Cell Therapeutics Medical Faculty, Heinrich‐Heine University Düsseldorf Germany; ^6^ Novo Nordisk Foundation Center for Stem Cell Medicine, Murdoch Children's Research Institute Melbourne VIC Australia; ^7^ Department of Clinical Pathology University of Melbourne Melbourne VIC Australia; ^8^ Australian Regenerative Medicine Institute Monash University Clayton VIC Australia; ^9^ Melbourne Centre for Cardiovascular Genomics and Regenerative Medicine Melbourne VIC Australia; ^10^ Cardiothoracic Surgery, Royal Children's Hospital Melbourne VIC Australia; ^11^ Department of Microbiology and Immunology Peter Doherty Institute for Infection and Immunity, University of Melbourne Melbourne VIC Australia; ^12^ Department of Paediatrics University of Melbourne Melbourne VIC Australia; ^13^ Department of Microbiology and Immunology University of Melbourne Melbourne VIC Australia

**Keywords:** CD38, flow cytometry, human immunology, invariant natural killer T cells, lymphocyte differentiation, NKT cells

## Abstract

Human invariant natural killer T cells (iNKT) play an important role in an orchestrated immune response; however, the heterogeneity of iNKT subsets is not yet fully understood. Here, we uncovered CD38 as a marker of iNKT differentiation, decoupling it from its role as a marker of activation by comparing the phenotype, cytokine profile and transcription factor expression of iNKT cell subsets in humans. Expression of CD38 on resting iNKT cells was restricted to cells that were low in well‐described maturity markers such as CD161 and CCR5 and co‐expressed markers associated with undifferentiated T cells (CD45RA, CCR7, CD62L). High abundance of CD38^+^ iNKT cells in human infant thymus and cord blood supported the immature nature of this subset. Functional analysis revealed that the CD38^+^ phenotype of resting iNKT cells was accompanied by diminished type 1 cytokine release, which was reflected by reduced expression of the transcription factor EOMES. Moreover, *in vitro* stimulation of sorted CD38^+^ and CD38^−^ iNKT cells demonstrated the distinct phenotype of cells expressing CD38 in a resting state and activation‐induced CD38. These findings suggest a context‐dependent role of CD38 expression on iNKT cells, distinguishing activated from resting iNKT cells where CD38 expression marks a subset of undifferentiated cells with altered functionality. Taken together, we describe a population of iNKT cells that extends the remarkable heterogeneity of the iNKT cell compartment beyond the presence of CD4^+^ and CD4^−^ subsets.

## Graphical abstract

In this study, we characterize a distinct subpopulation of undifferentiated invariant natural killer T (iNKT) cells in the peripheral blood of healthy adults, which are characterized by CD38 expression in a resting state. This CD38^+^ subset closely resembles iNKT cells from cord blood and thymus, exhibiting a unique cytokine profile and alterations in subset‐specific transcription factor expression.

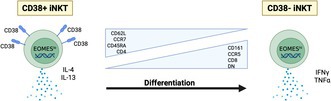

## INTRODUCTION

Invariant natural killer T (iNKT) cells are a group of unconventional T cells that express a semi‐invariant T‐cell receptor which in humans comprises the Vα24‐Jα18 rearrangement preferentially coupled to Vβ11. The invariant TCR facilitates recognition of exogenous and endogenous lipid antigens restricted to the nonclassical MHC class I‐like molecule CD1d, and the marine sponge derived glycolipid α‐Galactosylceramide (αGalCer) is considered the prototypic antigen for iNKT cells.^1^ Hallmarks of iNKT cells include their rapid effector response manifesting within hours following antigenic stimulation as well as their dual functionality. iNKT cells can directly lyse target cells; they also modulate downstream adaptive immune response by enhancing the maturation of dendritic cells and B cells, and they can release pro‐ and anti‐inflammatory cytokines rendering them a promising candidate for therapeutic applications.^2^, ^3^, ^4^, ^5^

Despite their invariant TCR, human iNKT cells display a remarkable subset heterogeneity. They can be classified into CD4^+^ and double‐negative (DN) cells in mice and humans while human iNKT cells comprise an additional CD8^+^ subset. Different effector functions have been attributed to the CD4^+^ and CD4^−^ subsets. Specifically, the CD4^+^ subset exhibits a multifunctional Th1/2‐like phenotype characterized by the production of IFNγ and IL‐4 upon stimulation, while CD4^−^ iNKT cells are polarized toward a Th1 effector function as they preferentially secrete IFNγ and mediate direct cytotoxicity.^6^, ^7^, ^8^ In addition, it has been shown that distinct human iNKT subsets can transactivate conventional T cells, B cells and NK cells which reflects their widespread potential to influence the downstream immune response.^9^ Other markers that group human iNKT cells into functional and phenotypical subsets have been discovered recently. The best described of those markers are CD161, which also acts as a maturation marker on iNKT cells, and CD62L which defines a subpopulation with superior longevity and enhanced antitumor functions.^10^, ^11^ The broad heterogeneity of human iNKT cells warrants further knowledge on subset differentiation and function to better understand the implications of these cells in human immunity.^7^, ^12^, ^13^, ^14^

iNKT cells are implicated in a wide range of diseases, such as cancer, autoimmunity and microbial infection.^12^, ^15^, ^16^, ^17^, ^18^ For instance, iNKT cells play a role in infections with human immunodeficiency virus (HIV), herpes simplex virus (HSV), influenza virus and in viral hepatitis.^16^ Along these lines, we recently described the involvement of human iNKT cells in acute and chronic hepatitis C virus (HCV) infection, in which activated human iNKT cells expressed CD38 in individuals infected with HCV. Moreover, CD38 was readily upregulated on iNKT cells from healthy donors after *in vitro* stimulation.^19^

CD38 is a multifaceted ectoenzyme expressed on a variety of immune cells. First reports of CD38 describe its expression on human thymocytes and its induction in Concanavalin A‐activated T lymphocytes resulting in a widespread use as a T‐cell activation marker.^20^, ^21^, ^22^ Indeed, many studies demonstrated the expression of CD38 on chronically activated T cells, especially in HCV and HIV infected patients.^23^, ^24^, ^25^ However, in recent years, a multitude of functions of CD38 in immune cells have been acknowledged such as migration, metabolism and cytokine secretion.^20^, ^26^ It was shown that expression of CD38 is not restricted to activated T cells but rather exhibits a subset‐specific role. In conventional T cells, CD38 is highly expressed on naïve cells with low metabolic activity, whereas its expression on activated effector T cells is associated with high cytokine secretion and immunopathology.^27^

In contrast to conventional T cells that exit the thymus in a naïve state and require priming in the periphery to fully differentiate into effector and memory cells, iNKT cells acquire a poised effector state in the thymus that facilitates immediate cytokine release.^1^, ^28^, ^29^ Despite this, most human iNKT cells appear to undergo further post‐thymic maturation, evidenced by increased frequencies of CD4^−^ cells in the periphery, accompanied by the expression of CD161 and CCR5.^10^, ^30^ Given the differential expression of CD38 on conventional naïve and effector T cells, elucidating the expression patterns of CD38 on human iNKT cell subsets might provide greater clarity about subset‐specific heterogeneity and function. This will lead to a better understanding of iNKT involvement in human disease as well as eventually inform decisions about iNKT subset use and stimulation protocols for therapeutic purposes.

Here, we reveal CD38 as a marker of an immature subset of iNKT cells with limited functional potential and identify a broader role of CD38 expression on iNKT cells that extends beyond activation.

## RESULTS

### Expression of CD38 on iNKT cells and conventional T cells from healthy donors

**Figure 1:**
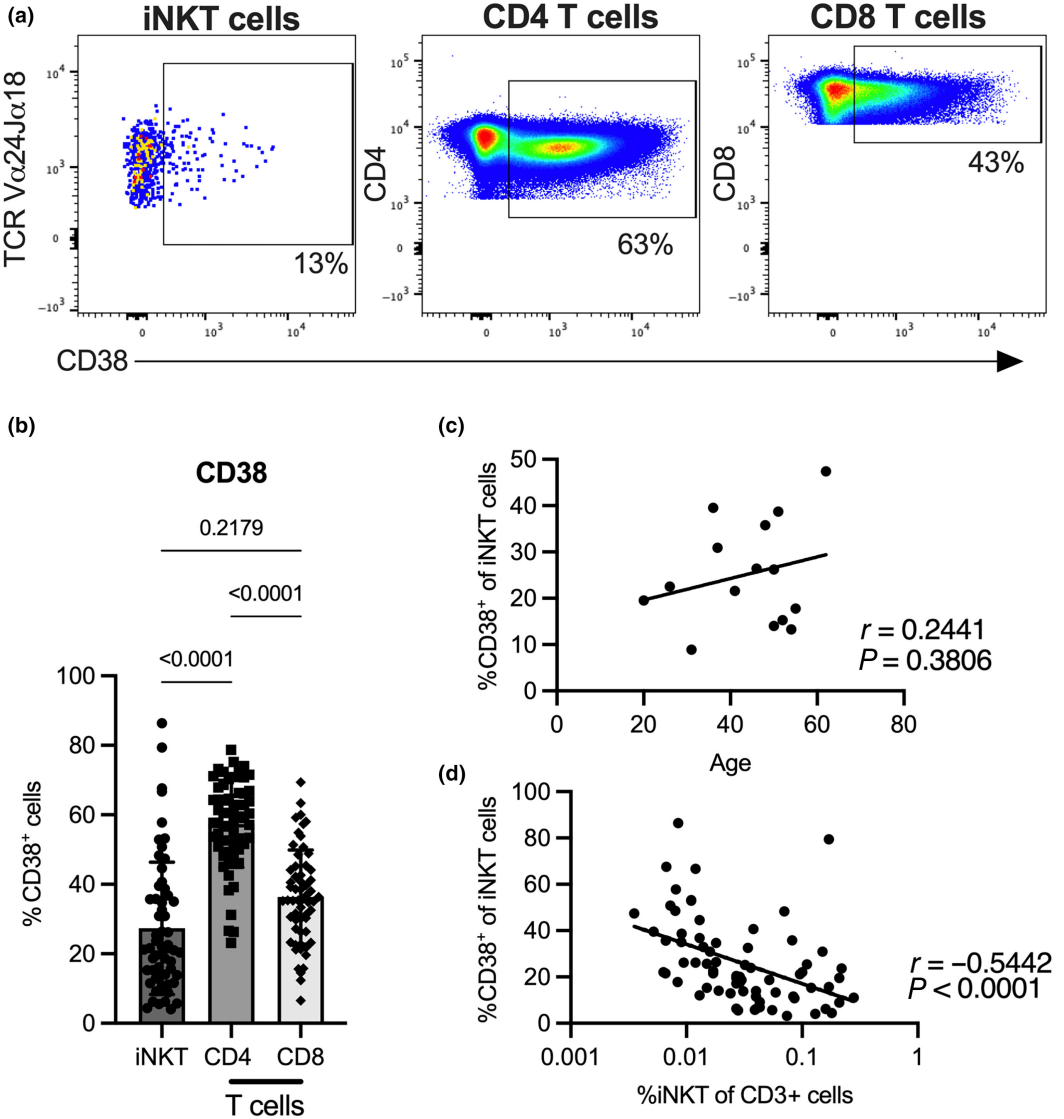
CD38 expression in healthy donor PBMC. **(a)** The expression of CD38 was analyzed on iNKT cells and on conventional CD4^+^ and CD8^+^ T cells from healthy donors. **(b)** Frequency of CD38 in the respective cell populations. Bar depicts the mean of *n* = 59 samples. Groups were compared with a Friedman test and the error bars depict the standard deviation. **(c)** Correlation of the frequency of CD38^+^ iNKT cells with age (*n* = 15) and **(d)** total iNKT cell frequency (*n* = 72) is shown. Correlations were calculated with Pearsons **(c)** or Spearmans **(d)** correlation analysis. Samples with 20 or fewer cells in one of the compared populations were excluded from the analysis.

In human immunological studies, the role of CD38 as a marker of activation is well described for conventional T cells; however, its expression and functional role in human iNKT cells has not been investigated in detail. We previously reported that iNKT cells upregulate CD38 after *in vitro* activation with αGalCer,^19^ but it is unclear if CD38 is also expressed on resting iNKT cells, independent from activation. Therefore, we first assessed the expression of CD38 on iNKT and conventional T cells from healthy adult donors in the absence of stimulation. A mean of 27% of iNKT cells expressed CD38 in a resting state, whereas a mean of 57% and 36% of all resting conventional CD4^+^ and CD8^+^ T cells were positive for this marker, respectively (Figure 1a, b). Although CD38 expression on iNKT cells did not correlate with donor age, it negatively correlated with total iNKT cell frequency in blood (Figure 1c, d).

### Expression of differentiation markers on resting CD38 ^+^ iNKT cells

**Figure 2:**
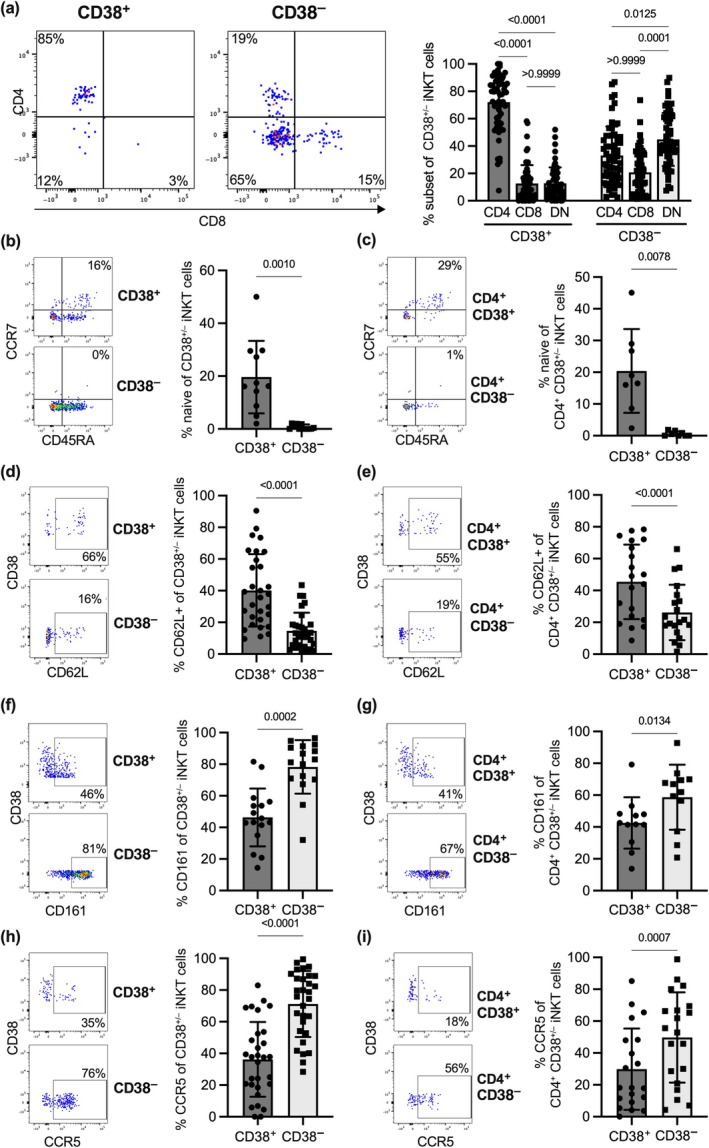
Comparison of the phenotype of CD38^+^ and CD38^−^ as wells as CD4^+^ CD38^+^ and CD4^+^ CD38^−^ iNKT cells. Healthy donor PBMC were gated into CD38^+^ iNKT cells and CD38^−^ iNKT cells **(a, b, d, f, h)** or gated on CD4^+^ iNKT cells and this subset was further gated into CD38^+^ and CD38^−^ cells **(c, e, g, i).** The expression of **(a)** CD4 and CD8 (*n* = 58), **(b, c)** co‐expression of CD45RA and CCR7 (double‐positive cells are referred to as naïve) (*n* = 11 and *n* = 8), **(d, e)** CD62L (*n* = 30 and *n* = 20), **(f, g)** CD161 (*n* = 16 and *n* = 12), and **(h, i)** CCR5 (*n* = 30 and *n* = 20) in the respective subsets is depicted as representative FACS plots (left panels) and bar graphs (right panels). Bars represent the mean, error bars depict the standard deviation and *P*‐values were calculated with a Friedman test **(a)**, a Wilcoxon test **(b, c, d, f, h, i)**, or a paired *t*‐test **(e, g)**. Samples with 20 or less cells in one of the compared populations were excluded from the analysis.

We next analyzed the phenotype of resting CD38^+^ iNKT cells compared with CD38^−^ iNKT cells in more detail (Figure 2). There was a strong enrichment of CD4^+^ iNKT cells in the CD38^+^ subset (mean 72%), whereas CD8^+^ and double‐negative (DN) cells were less frequent (mean 13% and 13%). In contrast, within the CD38^−^ subset, frequencies of CD4^+^ and CD8+ iNKT cells were not significantly different (mean 33% and 21%) and DN iNKT cells were moderately enriched (mean 45%; Figure 2a). The functional heterogeneity of CD4^+^ and CD4^−^ iNKT cells is well established.^6^, ^7^, ^12^, ^31^, ^32^, ^33^ Indeed, many of the markers we examined in this work were differentially expressed between CD4^+^ and CD4^−^ iNKT subsets (Supplementary figure 1).

iNKT cells are typically characterized by an effector/memory phenotype, which is reflected by low expression of CCR7 and CD45RA on iNKT cells.^30^, ^32^, ^34^ Although the majority of resting iNKT cells indeed lacked both CD45RA and CCR7 in our analysis, we could observe unexpectedly high numbers of iNKT cells that co‐expressed CD45RA and CCR7 in the CD38^+^ subset, which resembled a naïve T‐cell phenotype (Figure 2b). Here, a mean of 20% of CD38^+^ iNKT cells co‐expressed CD45RA and CCR7, whereas this phenotype was almost absent from the CD38^−^ subset (mean 0.7%) (Figure 2b).

Since the vast majority of CD38^+^ iNKT cells belong to the CD4^+^ subset (Figure 2a), we investigated whether the observed differences between CD38^+^ and CD38^−^ iNKT cells could be attributed to disparities between the CD4^+^ and CD4^−^ subset. However, even when gating on CD4^+^ cells before distinguishing between CD38^+^ and CD38^−^ populations, CD38 expression still marked iNKT cells with a CD45RA^+^CCR7^+^ naïve‐like phenotype (Figure 2c). This finding suggests that CD38^+^ iNKT cells constitute a distinct, less differentiated subpopulation independent of CD4 or CD8 expression.

Hence, we examined other markers associated with iNKT cell differentiation and maturation. In line with a less differentiated state, the CD38^+^ as well as the CD4^+^ CD38^+^ iNKT cell population were enriched for cells expressing CD62L (mean 40% *versus* 15% and 45% *versus* 26%, respectively), which is associated with a naïve or central memory phenotype in conventional T cells^35^ (Figure 2d, e). When compared with their CD38^−^ counterparts, there were fewer cells in the CD38^+^ and the CD4^+^ CD38^+^ subset expressing CD161 (Figure 2f, g; mean 46% *versus* 78% and 43% *versus* 59%, respectively) and CCR5 (Figure 2h, i; median 36% *versus* 71% and 30% *versus* 50%, respectively) – receptors which have been described to mark mature iNKT cells.^10^, ^30^ Moreover, additional markers associated with T‐cell differentiation and cytotoxicity such as KLRG‐1, CD56 and NKG2D were differentially expressed between CD38^+^ and CD38^−^ iNKT cells as well as between CD4^+^ CD38^+^ and CD4^+^ CD38^−^ iNKT cells (KLRG‐1) (Supplementary figure 2). Finally, cells expressing the chemokine receptor CCR2, but not CCR4 and CCR6, were less frequent in the CD38^+^ and CD4^+^ CD38^+^ iNKT cell subsets (Supplementary figure 2). Collectively, the data strongly indicate that in a resting state, the CD38^+^ subset represents a distinct, undifferentiated population of iNKT cells.

### Functional analysis of resting CD38^+^ iNKT cells

Having identified CD38 expression on resting iNKT cells as a marker for a phenotypically distinct subset, we next examined their functionality. To overcome the low abundance of iNKT cells in human peripheral blood, iNKT cells were magnetically enriched followed by *in vitro* stimulation and intracellular cytokine staining. Prior to analysis, the kinetics of CD38 expression on iNKT cells upon stimulation was thoroughly assessed to ensure that only cells were included in our analysis that expressed CD38 in the resting state and not as the consequence of stimulation (Supplementary figure 3). Stimulation of enriched iNKT cells with PMA and ionomycin induced a profound upregulation of IFNγ, TNFα, IL‐2, IL‐4 and IL‐13 across all donors (Supplementary figure 4). Consistent with their well‐described Th1/Th2 phenotype, CD4^+^ iNKT cells produced IFNγ, TNFα and IL‐2, as well as the Th2 cytokines IL‐4 and IL‐13, but only marginal amounts of Granzyme B (Supplementary figure 5). DN and CD8^+^ iNKT cells showed a pronounced bias toward a type 1 cytokine profile as they secreted IFNγ and TNFα at comparable levels to CD4^+^ iNKT cells, and high levels of Granzyme B in the CD8^+^ subset, but produced only minimal IL‐4 and IL‐13 (Supplementary figure 5).

**Figure 3:**
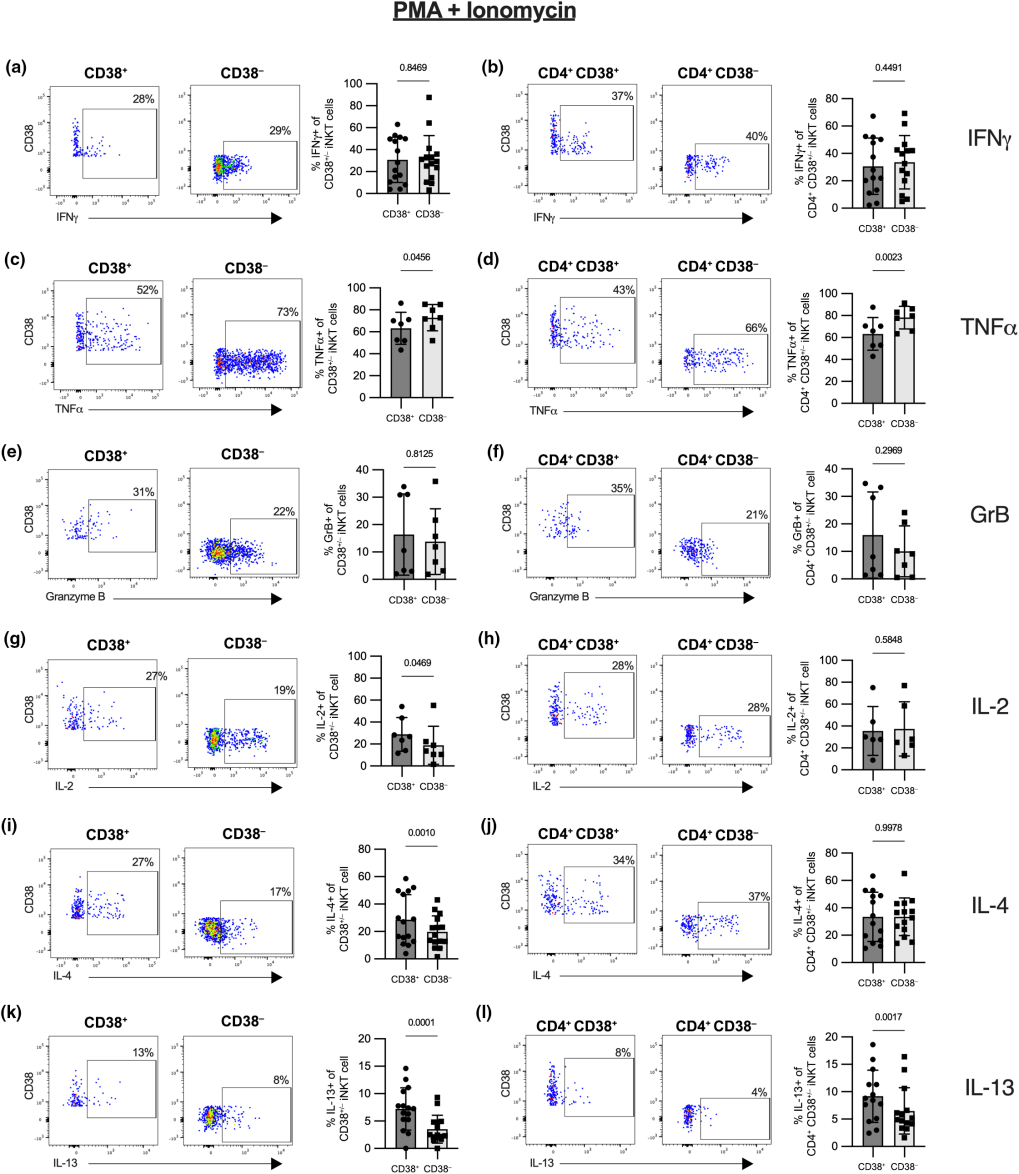
Functionality of CD38^+^ and CD38^−^ iNKT cells as well as CD4^+^ CD38^+^ and CD4^+^ CD38^−^ iNKT cells after stimulation with PMA and ionomycin. MACS‐enriched iNKT cells from healthy donor PBMC were stimulated with PMA and ionomycin for 6 h in the presence of BFA for the last 4 h. The production of **(a, b)** IFNγ (*n* = 15 and *n* = 14), **(c, d)** TNFα (*n* = 7 and *n* = 7), **(e, f)** Granzyme B (*n* = 7 and *n* = 7), **(g, h)** IL‐2 (*n* = 7 and *n* = 6), **(i, j)** IL‐4 (*n* = 15 and *n* = 14), and **(k, l)** IL‐13 (*n* = 15 and *n* = 14) was analyzed by flow cytometry in CD38^+^ and CD38^−^ **(a, c, e, g, i, k)** or CD4^+^ CD38^+^ and CD4^+^ CD38^−^ **(b, d, f, h, j, l)** iNKT cells. Representative FACS plots are shown in the left panels and bar graphs in the right panels. Bars represent the mean, the error bars show the standard deviation and a Wilcoxon test **(a, e, f, g, k, l)** or paired *t*‐test **(b, c, d, h, i, j)** was used for statistical testing. Samples with 20 or less cells in one of the compared populations were excluded from the analysis.

With the exception of a marginal reduction in TNFα secretion, we found no apparent difference in Th1 immunity – as indicated by IFNγ, TNFα, or Granzyme B expression – between CD38^+^ and CD38^−^, as well as CD4^+^ CD38^+^ and CD4^+^ CD38^−^ iNKT cells (Figure 3a–f). While there was a significant increase in IL‐2, IL‐4 and IL‐13 in CD38^+^ iNKT cells compared with CD38^−^ iNKT cells (Figure 3g, i, k), there was no difference in the secretion of IL‐2 and IL‐4 and just a very slight, albeit significant, difference in the secretion of IL‐13 between these populations within the CD4^+^ iNKT subset (Figure 3h, j, l). This suggests that the disparities observed between the CD38^+^ and CD38^−^ subsets were partially associated with CD4 expression rather than differential CD38 expression.

**Figure 4:**
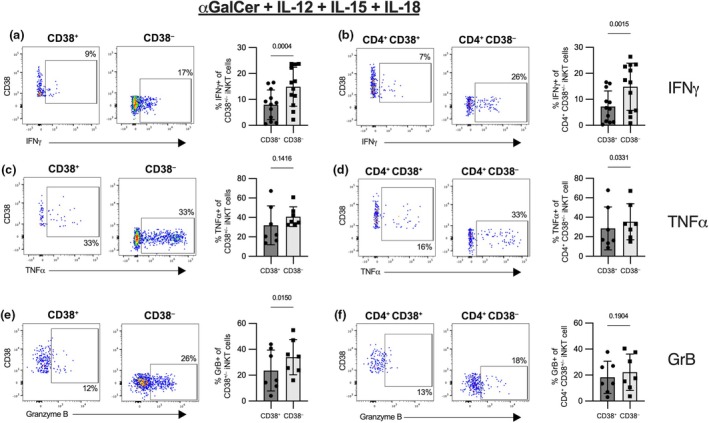
Functionality of CD38^+^ and CD38^−^ iNKT cells as well as CD4^+^ CD38^+^ and CD4^+^ CD38^−^ iNKT cells after stimulation with αGalCer and IL‐12, IL‐15 and IL‐18. MACS‐enriched iNKT cells from healthy donor PBMC were stimulated with αGalCer and IL‐12, IL‐15 and IL‐18 for 8 h in the presence of BFA for the last 4 h. The production of **(a, b)** IFNγ (*n* = 12 and *n* = 12), **(c, d)** TNFα (*n* = 7 and *n* = 7), or **(e, f)** Granzyme B (*n* = 7 and *n* = 7) was analyzed by flow cytometry in CD38^+^ and CD38^−^ **(a, c, e)** or CD4^+^ CD38^+^ and CD4^+^ CD38^−^ **(b, d, f)** iNKT cells. Representative FACS plots are shown in the left panels and bar graphs in the right panels. Bars represent the mean, error bars show standard deviation, and paired *t*‐test was used for statistical testing. Samples with 20 or fewer cells in one of the compared populations were excluded from the analysis.

Next, we employed a modified version of a protocol we had previously established that elicits high IFNγ secretion following stimulation of human iNKT cells with αGalCer and IL‐12, IL‐15 and IL‐18.^19^ Even though the overall cytokine production appeared lower than with PMA and ionomycin stimulation, we detected reasonable levels of IFNγ, TNFα and high amounts of Granzyme B (Supplementary figure 4). Using this stimulus, we observed significantly diminished levels of IFNγ and Granzyme B, but not TNFα, in CD38^+^ iNKT cells compared with CD38^−^ iNKT cells (Figure 4a, c, e). Furthermore, we observed about a 50% reduction in IFNγ secretion (mean 7.2% *versus* 14.8%) and a slight yet significant decrease in TNFα production in the CD4^+^ CD38^+^ compared with the CD4^+^ CD38^−^ population with no difference in Granzyme B levels (Figure 4b, d, f). This reveals diminished Th1 immunity in CD38^+^ NKT cells, especially the potential to secrete IFNγ. As this protocol was optimized for the secretion of IFNγ, other cytokines, such as IL‐2, IL‐4 and IL‐13 were detectable only at low levels (Supplementary figure 4) and did not show any significant differences between the two CD38 subpopulations (data not shown). By dividing the CD38^+^ population based on CCR7 and CD45RA, we could identify additional heterogeneity in the CD38^+^ iNKT cell population. iNKT cells co‐expressing CD38 along CD45RA and CCR7 – considered iNKT cells with a naïve phenotype – were clearly the most immature cells as demonstrated by the lack of CD161 expression and IFNγ secretion (Supplementary figure 6). The CD38^−^ iNKT cell population was almost completely devoid of CCR7 and CD45RA expressing cells (Figure 2b) whereas CD38^+^ iNKT cells also comprised cells that were negative for CCR7 and CD45RA (Figure 2b). These cells responded to PMA and ionomycin stimulation and secreted IFNγ at levels comparable to their CD38^−^ counterparts. When stimulated with αGalCer and IL‐12, IL‐15 and IL‐18; however, CD38^+^ CCR7^−^CD45RA^−^ cells had a diminished production of IFNγ compared with CD38^−^ iNKT cells indicating that the loss of CCR7 and CD45RA expression is the first step toward functional maturity (Supplementary figure 6).

Overall, our functional data show that resting CD38^+^ iNKT cells have a reduced capacity to secrete type 1 cytokines in response to antigenic stimulation, suggesting these cells are functionally immature.

### Transcription factor expression of CD38^+^ iNKT cells

The differential cytokine production between CD38^+^ and CD38^−^ iNKT cells further supports the existence of a distinct iNKT subpopulation with altered functionality defined by CD38 expression. To investigate whether these functional differences correspond to transcriptional variations, we analyzed the expression of key transcription factors associated with the iNKT lineage and various polarized T helper subsets previously described in murine iNKT cells.^36^, ^37^ Interestingly, significant differences in the expression levels of transcription factors were evident among human iNKT cell subsets. While CD4^+^ iNKT cells expressed the highest levels of PLZF, their expression of EOMES and RORγt was reduced compared with the CD4^−^ populations (Supplementary figure 7). The CD4^−^ subsets appeared to be relatively homogeneous with the exception of T‐bet expression, which was elevated in CD8^+^ but not in DN iNKT cells compared with the CD4^+^ subset, and GATA3, which was expressed slightly more in the DN subset (Supplementary figure 7).

**Figure 5:**
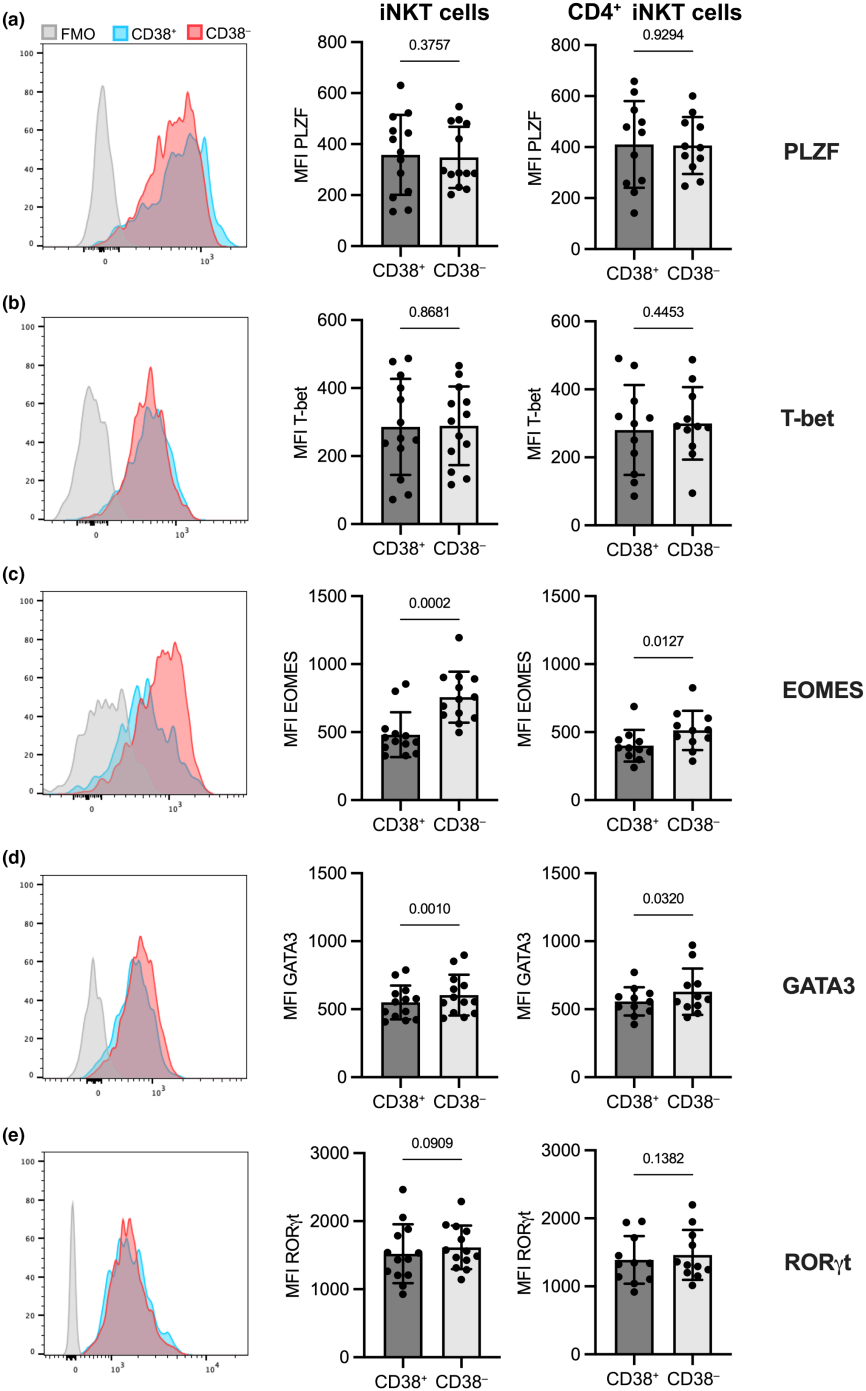
Transcriptional status of CD38^+^, CD38^−^, CD4^+^ CD38^+^ and CD4^+^ CD38^−^ iNKT cells. Transcription factor expression of healthy donor PBMC was analyzed by flow cytometry. Cells were gated on CD38^+^ and CD38^−^ iNKT cells (left and middle panel) or on CD4^+^ cells and this subset was further gated into CD38^+^ and CD38^−^ cells (right panel). The median fluorescence intensity of **(a)** PLZF; **(b)** T‐bet; **(c)** EOMES; **(d)** GATA3; and **(e)** RORγt in the respective subsets is shown. Representative histograms for the stained CD38^+^ iNKT population, the stained CD38^−^ iNKT population, and the fluorescence minus one (FMO) control of the total iNKT population is shown in the left panels. Bars represent the mean of *n* = 13 in the middle panels and *n* = 11 in the right panels and error bars represent the standard deviation. Groups were compared with a Wilcoxon test (**a** middle panel, **c** middle panel) or paired *t*‐test (**a** right panel, **b, c** right panel, **d, e**). Samples with 20 or fewer cells in one of the compared populations were excluded from the analysis.

When we compared CD38^+^ and CD38^−^ iNKT cells, we found no differences in the expression of the key iNKT cell transcription factor PLZF between these groups, neither in bulk iNKT cells nor within the CD4^+^ CD38^+^ subset (Figure 5a). While T‐bet was unaltered between the CD38^+^ and CD38^−^ subpopulations (Figure 5b), EOMES, another transcription factor associated with Th1 polarization and cytotoxicity, was significantly lower in CD38^+^ and CD4^+^ CD38^+^ iNKT cells compared with the corresponding CD38^−^ and CD4^+^ CD38^−^ populations (Figure 5c). The Th2 transcription factor GATA3 showed a slight yet significant decrease in the CD38^+^ and CD4^+^ CD38^+^ cells, while the Th17 transcription factor RORγt remained unchanged between groups (Figure 5d, e). In conclusion, the low expression of EOMES in CD38^+^ iNKT cells may account for their diminished ability to secrete IFNγ after αGalCer stimulation.

### Comparison of resting and activated CD38^+^ iNKT cells

**Figure 6:**
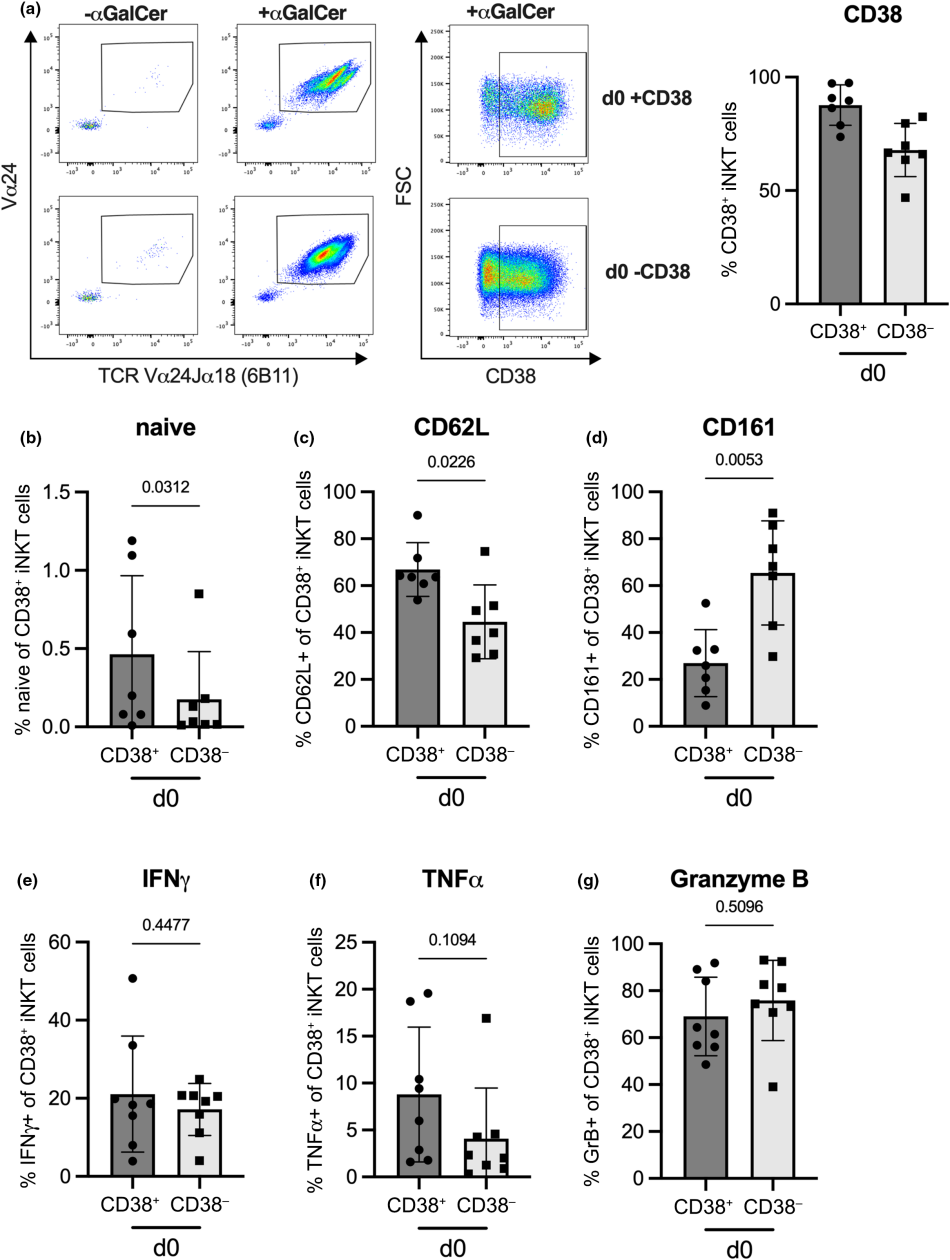
Phenotype of *in vitro* expanded CD38^+^ and CD38^−^ iNKT cells. CD38^+^ and CD38^−^ iNKT cells were sorted from healthy donor PBMC and cultured *in vitro* in the presence or absence of αGalCer and IL‐2 for 2 weeks. **(a)** The expansion of iNKT cells and the expression of CD38 after 2 weeks of culture is depicted by representative FACS plots and the bar graph shows the CD38 expression after expansion with αGalCer. The expression of **(b)** CD45RA and CCR7 (naïve), **(c)** CD62L and **(d)** CD161 on iNKT cells that were CD38^+^ after 2 weeks of αGalCer expansion was analyzed by flow cytometry. The production of **(e)** IFNγ, **(f)** TNFα, **(g)** Granzyme B was analyzed after restimulation with aGalCer, IL‐12, IL‐15 and IL‐18 following the 2 weeks αGalCer culture on iNKT cells that were CD38^+^ post expansion. Bars represent the mean of *n* = 7 **(a–d)** and *n* = 8 **(e–f)** donors, error bars depict the standard deviation, and each dot represents the average of one to three independent experiments per donor. A paired *t*‐test **(c, d, e, g)** or Wilcoxon test **(b, f)** was used for statistical analysis.

Given the widespread use of CD38 as an activation marker, we conducted a comparative analysis between the phenotype of *ex vivo* CD38^+^ iNKT cells and *ex vi*vo CD38^−^ iNKT cells after *in vitro* stimulation and upregulation of CD38. INKT cells were sorted into CD38^+^ and CD38^−^ cells and expanded *in vitro* with αGalCer. This resulted in a massive expansion of both CD38^+^ as well as CD38^−^ iNKT cells, accompanied by the upregulation of CD38 in the latter (Figure 6a). We then examined CD38^+^ iNKT cells in the cultures that already expressed CD38 *ex vivo* (d0 CD38^+^) and in the cultures that were initially CD38‐negative but induced its expression upon *in vitro* stimulation (d0 CD38^−^). Remarkably, despite the cells expressing CD38 after *in vitro* stimulation, the differences observed *ex vivo* partially persisted after *in vitro* expansion. Induced CD38^+^ iNKT cells (d0 CD38^−^) were still lower in CD62L while they exhibited higher amounts of CD161 (Supplementary figure 8, Figure 6c, d). In contrast, cells with a naïve‐like phenotype were virtually undetectable after *in vitro* stimulation (Supplementary figure 8, Figure 6b). This underscores that resting CD38^+^ iNKT cells are fundamentally different from iNKT cells expressing CD38 in the context of activation as they remain phenotypically distinct even after *in vitro* activation. Functional analysis of *in vitro* expanded iNKT cells revealed no striking difference between d0 CD38^+^ and d0 CD38^−^ iNKT cells (Figure 6e–g). The restored ability of CD38^+^ cells to produce IFNγ following *in vitro* expansion suggests a gain in functional competence after antigen encounter that could reflect a physiological process in the periphery.

### Analysis of CD38 expression on human thymic and cord blood iNKT cells

**Figure 7:**
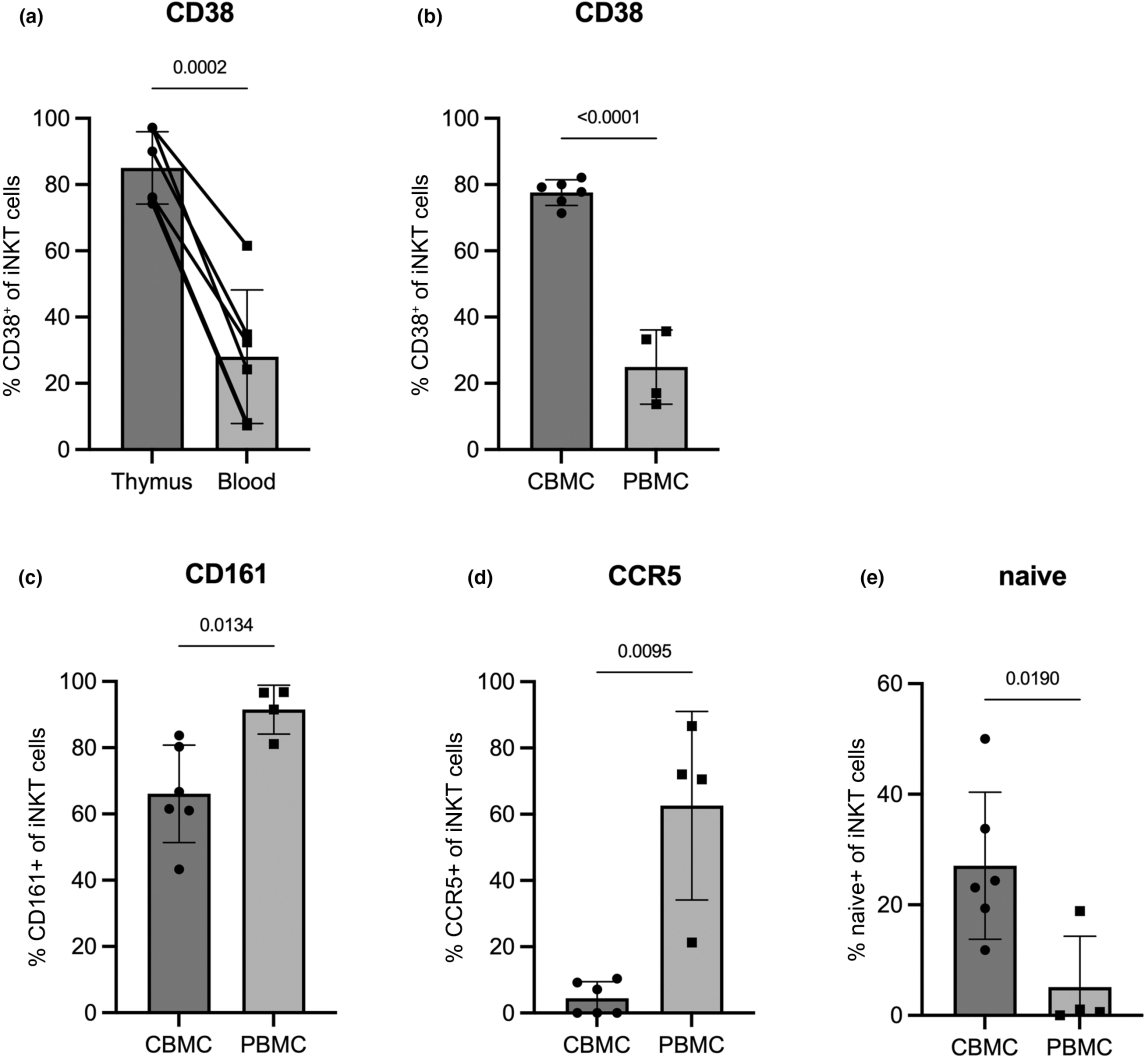
CD38 is expressed on thymus and cord blood iNKT cells. **(a)** The expression of CD38 was analyzed on iNKT cells from human infant thymus and matched peripheral blood. Percentage of **(b)** CD38, **(c)** CD161, **(d)** CCR5 and **(e)** CD45RA and CCR7 (naïve) on iNKT cells from human cord blood or adult PBMC. Bar depicts the mean of **(a)** *n* = 6 donors for thymus and matched blood samples or **(b–e)** *n* = 6 and *n* = 4 donors for cord blood and adult PBMC, respectively. Error bars show the standard deviation and significance was tested with a paired *t*‐test **(a)**, unpaired *t*‐test **(b, c)**, or Mann–Whitney test **(d, e)**.

To confirm the link between iNKT immaturity and CD38 expression, we analyzed CD38 on iNKT cells from human infant thymic tissue as well as from cord blood. CD38^+^ iNKT cells were strongly enriched in human thymocytes compared with matched PBMC (Figure 7a) as well as in cord blood mononuclear cells (CBMC) compared with adult PBMC (Figure 7b). A higher frequency of iNKT cells in cord blood exhibited a naïve‐like phenotype characterized by the expression of CD45RA and CCR7 compared with adult PBMC while significantly reduced numbers of cord blood iNKT cells expressed CCR5 and CD161 (Figure 7c–e). Thus, immature thymic and cord blood iNKT cells strongly resembled the phenotype we observed in CD38^+^ iNKT cells from adult PBMC providing further evidence that CD38^+^ NKT cells represent an immature, functionally distinct subset of iNKT cells.

Overall, *ex vivo* CD38 expression on resting human iNKT cells is associated with an undifferentiated phenotype, observed both in bulk iNKT cells and within the CD4^+^ subset. Moreover, CD38^+^ iNKT cells are highly abundant in human infant thymus and cord blood. These cells exhibit a reduced capacity for IFNγ production upon antigenic stimulation, potentially due to lower expression of the Th1 transcription factor EOMES. While CD38 remains a reliable marker of iNKT cell activation, as evidenced by its upregulation on CD38^−^ cells following *in vitro* stimulation, its expression under steady‐state conditions likely defines a distinct subset of immature iNKT cells that partially retain their unique phenotype even after activation.

## DISCUSSION

Human iNKT cells exhibit a plethora of different functions and are involved in a variety of human pathologies including microbial infections, which makes them an attractive therapeutic target.^2^ Despite their invariant TCR, these cells exhibit notable subset heterogeneity, with distinct and sometimes opposing effector functions. A comprehensive understanding of human iNKT cell subsets beyond the traditional CD4^+^ and CD4^−^ classification is essential for harnessing their potential in immunotherapy or as vaccine adjuvants.^7^, ^38^ In this line, CD62L^+^ iNKT cells have been demonstrated to possess superior antitumor potential regardless of CD4 expression.^11^ In this study, we describe an iNKT cell subpopulation with a distinct phenotype, function and transcriptional status based on the expression of CD38 in the resting state.

It has been shown that CD4^+^ iNKT cells appear more immature than CD4^−^ iNKT cells and can be further subdivided based on CCR5 expression.^30^ Here, we demonstrate that the expression of CD38 on CD4^+^ iNKT cells of healthy donors is linked to an immature phenotype. This was indicated by the co‐expression of CCR7 and CD45RA alongside CD62L as well as the reduced expression of markers associated with iNKT and T‐cell differentiation such as CCR5, CD161 and KLRG‐1.^30^, ^39^, ^40^, ^41^, ^42^

Even though iNKT cells leave the thymus as poised effector cells, they undergo a certain degree of peripheral maturation. One of the best‐defined maturation markers for human iNKT cells is CD161. Many iNKT cells in the thymus and cord blood are CD161^−^, and CD161^+^ iNKT cells accumulate in peripheral blood with age. Thereby, the upregulation of CD161 is believed to be an important key event during iNKT maturation which likely occurs in the periphery.^40^, ^43^, ^44^ Here, we detected high levels of CD38 in iNKT cells from postnatal thymus and cord blood but lower levels in PBMC from infants and adults. The lower expression of CD38 in pediatric blood samples compared with cord blood indicates that maturation of iNKT cells begins early in life but is not established prior to birth. This downregulation of CD38 after thymic egress corresponds with increased expression of CD161 on iNKT cells in matched blood compared with postnatal thymus.^43^

Given the strong evidence for CD161 as a marker for iNKT maturation and the recently described lack of CCR5 expression on immature iNKT cells,^30^ the opposing expression of CD38 with CD161 and CCR5 in our study proposes CD38 as a marker for an immature peripheral subpopulation of human iNKT cells.

Using TCR sequencing and clonotype analysis, Liu *et al*. observed that peripheral CD4^+^ iNKT cells give rise to more differentiated, CD4^−^ iNKT cells *in vivo*. However, this could not be fully recapitulated *in vitro*, potentially due to the lack of an unknown differentiation signal.^30^ Even though the majority of thymic and cord blood iNKT cells expressed CD38 in our analysis, a small number of CD38^−^ cells were also detected. Whether peripheral CD38^−^ iNKT cells arise from CD38^+^ precursors in response to a yet unknown *in vivo* signal in the periphery or if these cells emigrate from the thymus as two separate lineages remains to be elucidated in future studies.

Akin to the well documented functional differences among iNKT subsets with respect to the expression of CD4, CD62L or CD161,^6^, ^7^, ^10^, ^31^, ^45^ CD38 expression was associated with diminished secretion of Th1 cytokines in our study. Specifically, in the most immature CD4^+^ iNKT subset, CD38^+^ iNKT cells responded with weaker IFNγ secretion to antigen‐specific stimulation. Baev *et al*.^46^ reported that thymic and cord blood human iNKT cells are not fully mature, are almost exclusively CD4^+^, and do not respond to *in vitro* anti‐CD3 stimulation without several rounds of expansion. Chan *et al*.^10^ later confirmed this immature phenotype of human thymic and cord blood iNKT cells, amended by the observation of profound cytokine production after PMA and ionomycin stimulation. This strikingly resembles what we observed for CD4^+^ CD38^+^ iNKT cells in the periphery of adult donors which were phenotypically immature and had impaired IFNγ production after antigenic but not PMA and ionomycin stimulation indicating that CD38^+^ iNKT cells have not acquired their full functional potential yet. Interestingly, upon *in vitro* expansion with the prototypic iNKT cell ligand αGalCer the functional differences between the two subsets disappeared, suggesting a gain of functional competence of CD38^+^ iNKT cells during prolonged culture in the presence of antigen. Whether endogenous signals are sufficient to induce this differentiation event that fully unlocks the functional potential of iNKT cells *in vivo* or if it relies on exogenous ligands, for example, interaction with the commensal microbiota, invites for speculation.^47^, ^48^

The decreased expression of the transcription factor EOMES in CD38^+^ iNKT cells may provide a possible mechanism for the observed reduction in IFNγ production. While the differential expression of the key transcription factors PLZF, T‐bet, GATA3 and RORγt in NKT1, NKT2 and NKT17 cells is established in mice,^37^, ^49^ these subsets have not been clearly identified in humans.^38^, ^50^ The extent to which human iNKT cell subsets follow the same transcriptional regulation as mouse iNKT cells remains unclear. Therefore, it is intriguing that the diminished IFNγ secretion observed in human CD38^+^ iNKT cells coincides with decreased EOMES but not T‐bet expression. EOMES has been shown to be crucial for the development and effector function of human NK cells^51^, ^52^ and its expression has been linked to IFNγ secretion in a subset of human nonclassical Th1 cells.^53^ In a conditional knockout mouse model, EOMES was critical for the generation of NKT1 cells and for the differentiation of iNKT cells into KLRG‐1 expressing memory‐like cells.^54^ Our observation of reduced NKT1 effector function and KLRG‐1 expression, alongside decreased EOMES expression in the CD38^+^ subset, suggests a similar involvement of EOMES in the differentiation of human iNKT cells.

Consistent with our findings, one study reports a difference in T‐bet and EOMES expression in CD4^+^ compared with CD4^−^ iNKT cells.^55^ While this difference was mostly based on varying frequencies of cells positive for each transcription factor, our study corroborates these findings showing varying expression levels between CD4^+^ and CD4^−^ iNKT cells based on the MFI. Additionally, we refine this distinction within the CD4^−^ subset, highlighting that while DN and CD8^+^ iNKT cells express similar levels of EOMES, the CD8^+^ iNKT subset exhibits the highest levels of T‐bet. Overall, these data indicate a pivotal role for EOMES in the regulation of human iNKT cell functionality.

While the role of CD38 on human iNKT cells had not been explored previously, other studies have investigated CD38 expression on conventional T cells. In accordance with our data, CD38 expression on conventional T cells has been linked to a diminished IFNγ production in several studies.^56^, ^57^, ^58^ Besides these functional implications, it has been described very early on that expression of CD38 is not limited to activated T cells but is present on CD45RA expressing naïve T cells.^59^ More recently, it has been shown that CD38 is expressed on almost all naïve T cells under homeostatic conditions and that these naïve CD38^+^ T cells lack effector functions, while CD38 upregulation after activation is restricted to effector T cells where its expression is associated with increased cytokine expression.^27^ Mechanistically, the authors described a role of CD38 in the reduction of intracellular NAD^+^ levels and the dampening of naïve T‐cell metabolism. Furthermore, high expression of CD38 emerged as a novel marker for recent thymic emigrants (RTE) on human conventional CD4^+^ and CD8^+^ T cells.^60^ While the mechanistic role of CD38 expression on human iNKT remains unclear, the many parallels between CD38 expression on naïve T cells and the immature phenotype of CD38^+^ iNKT cells suggest a similar role of CD38 on iNKT cells.

Overall, expression of CD38 on human resting peripheral blood iNKT cells is associated with an immature phenotype and a reduced Th1 functionality that is reflected at a transcriptional level. High abundancy of CD38‐expressing iNKT cells in the infant thymus and in cord blood confirmed the immaturity of this cell subset. These differences persist after *in vitro* activation, suggesting novel roles of CD38 in human iNKT cell subset heterogeneity that reach beyond T‐cell activation.

## METHODS

### Preparation of human samples

Blood from healthy adult donors was collected with informed consent and ethical approval from the local ethics committee (#3240) at the University Hospital Düsseldorf's Centre for Blood Donation, and at the Australian Red Cross Lifeblood under agreement number 23‐06VIC‐01. Cord blood was provided by the Royal Children's Hospital, Melbourne, Australia, with ethics approval from the Royal Children's Hospital Melbourne Human Research Ethics Committee (HREC24131). Neonatal and pediatric thymus (aged 4–282 days) and whole blood samples were taken from consented cardiac surgery patients (HREC38192) and obtained from the Melbourne Children's Heart Tissue Bank (MCHTB) for this analysis. Peripheral blood mononuclear cells (PBMC) and cord blood mononuclear cells (CBMC) were isolated by Ficoll‐Paque density gradient centrifugation and cryopreserved in 90% FBS (Biochrome, Cambridgeshire, UK) and 10% dimethyl sulfoxide (Roth, Karlsruhe, Germany). Donor thymi were cut into small pieces and passed through a 70‐μm filter into PBS containing 2% FBS and washed twice, prior to magnetic bead enrichment. Single‐cell suspensions of human thymus were enriched for iNKT cells by staining with anti‐Vα24Jα18 (6B11) PE‐Cy7 antibody followed by magnetic bead enrichment using anti‐PE microbeads and LS columns as per manufacturer's instructions (Miltenyi, Bergisch Gladbach, Germany).

### Flow cytometry analysis

All antibodies, staining reagents and flow cytometry panels are summarized in Supplementary tables 1 and 2. Cells were washed twice in DPBS (Gibco, Thermo Fisher Scientific, Waltham, MA, USA) and stained with fixable viability dye (FvD) in PBS for 15 min at 4°C. After a wash with FACS buffer (PBS + 2% FCS) cells were stained with surface antibodies in FACS buffer and BD Brilliant Stain buffer (BD Bioscience, Franklin Lakes, NJ, USA) for 15 min at 4°C. Human iNKT cells were identified with an antibody against the invariant TCR alpha chain Vα24‐Jα18 (clone 6B11) and in experiments that required a high specificity to identify iNKT cells, a separate antibody against the Vα24 TCR chain was used. iNKT cells were gated as FvD^−^CD14^−^CD19^−^CD3^+^Va24^+^6B11^+^ cells (Supplementary figure 9). Subsequently, all cells were washed with FACS buffer and fixed with IC fixation buffer (Thermo Fisher Scientific, Waltham, MA, USA) for 15 min at 4°C before analysis or intracellular staining. For intracellular cytokine staining, fixed cells were washed twice with 1× permeabilization buffer (Thermo Fisher Scientific) and antibodies against intracellular antigens added in 1× permeabilization buffer for 20 min at 4°C. Transcription factors were stained following the surface staining with the Foxp3/Transcription Factor Staining Buffer Set (Thermo Fisher Scientific) according to the manufacturers protocol. Samples were washed with FACS buffer and acquired on a BD LSRFortessa or a Cytek Aurora and analyzed with FlowJo software. Samples with <20 iNKT cells were excluded from the analysis.

### *In vitro* stimulation of iNKT cells

Cryopreserved PBMC were thawed, washed twice with DPBS (Gibco, Thermo Fisher Scientific) and seeded in a concentration of 2 × 10^6^ cells mL^−1^ in R10 (RPMI1640 + 10% FCS + 1% Penicillin/Streptomycin). Cells were rested overnight at 37°C, 5% CO_2_ in a humidified incubator before they were stained with 6B11 PE antibody and magnetically enriched using anti‐PE microbeads (Miltenyi, Bergisch Gladbach, Germany) and LS columns (Miltenyi) according to the manufacturer's instructions. The concentration of the enriched iNKT cells was adjusted to 2 × 10^6^ cells mL^−1^ using the iNKT negative fraction and seeded into cell culture plates to facilitate activation. Enriched iNKT cells were stimulated with 10 ng mL^−1^ PMA and 1 μg mL^−1^ ionomycin for 6 h, or a combination of 1 μg mL^−1^ aGalCer, 10 ng mL^−1^ IL‐12, 100 ng mL^−1^ IL‐15 and 50 ng mL^−1^ IL‐18 for 8 h. Brefeldin A (BFA) was added to the cultures in a concentration of 100 ng mL^−1^ for the last 4 h before cells were harvested and analyzed by flow cytometry.

### *In vitro* expansion of iNKT cells

Sorted iNKT cells were expanded in the presence of autologous feeder cells from the same donor. For the preparation of feeder cells, cryopreserved PBMC were thawed and iNKT cells depleted with iNKT microbeads (Miltenyi) and LD columns (Miltenyi). The flow through was washed and cultured in R10 in a concentration of 2 × 10^6^ cells mL^−1^. The next day the cells were irradiated with 30 Gray and half of the cells pulsed with 1 μg mL^−1^ αGalCer for at least 1 h. Sorted CD38^+^ and CD38^−^ iNKT cells were cocultured with pulsed or unpulsed feeder cells in R10 supplemented with 25 U mL^−1^ IL‐2. Additionally, 1 μg mL^−1^ αGalCer was added to the pulsed cultures. After five to 7 days, half of the initial volume of fresh R10 + 25 U mL^−1^ IL‐2 was added to the cultures.

### Fluorescence activated cell sorting

PBMC were thawed and rested overnight in R10 in a concentration of 2 × 10^6^ cell mL^−1^. The next day, cells were stained with FvD eF780, CD14 APC‐eF780, CD19 APC‐eF780, 6B11 PE and CD38 APC. Monocytes and B cells were excluded from viable PBMC by CD14 and CD19. iNKT cells were identified by TCR Va24‐Ja18 (clone 6B11) staining followed by sorting into a CD38^+^ and CD38^−^ iNKT cell population (Supplementary figure 10). Approximately, 20 million stained PBMC were sorted, and recovery of iNKT cells ranged from at least 100 up to almost 5000 cells. The purity of all sorted cells was verified to be at least 90% in a postsort analysis. Cells were sorted on a MoFlo XDP cell sorter (BeckmanCoulter) in the flow cytometry and cell sorting facility of the Institute for Transplantation Diagnostics and Cell Therapeutics at the University Hospital Düsseldorf, Germany.

### Statistical analysis

GraphPad Prism (version 10.0.3 for MacOS, GraphPad Software, Boston, MA, USA) software was used for figure generation and statistical analyses. Data were tested for normality with a Shapiro–Wilk test and a parametric or nonparametric test was chosen based on the normal distribution of the data. For comparisons between two groups, a paired *t*‐test or Wilcoxon matched‐pairs signed‐ranks test were used for normally or not normally distributed data, respectively. An unpaired *t*‐test (parametric) or a Mann–Whitney test (nonparametric) was applied when comparing unpaired data sets. Comparisons involving three or more groups were conducted using a one‐way ANOVA for normally distributed or a Kruskal–Wallis test for not normally distributed data. Correlations were assessed using either Pearson's test (parametric) or Spearman's test (nonparametric). A *P*‐value of ≤0.05 was considered statistically significant.

## AUTHOR CONTRIBUTIONS

**Christopher Menne:** Conceptualization; investigation; writing – original draft; formal analysis; visualization. **Naeimeh Tavakolinia:** Investigation; formal analysis; visualization. **Louis Perriman:** Formal analysis; visualization. **Wiebke Moskorz:** Writing – review and editing; investigation. **Christine Cosmovici:** Conceptualization. **Andreas Walker:** Conceptualization. **Lara Olejnik:** Writing – review and editing; investigation. **Katharina Raba:** Investigation; methodology. **Mei RM Du:** Formal analysis; visualization. **Fernando J Rossello:** Formal analysis; visualization. **Igor E Konstantinov:** Resources. **Stuart P Berzins:** Resources. **Daniel G Pellicci:** Conceptualization; writing – review and editing; supervision. **Jörg Timm:** Conceptualization; writing – original draft; supervision; funding acquisition.

## CONFLICT OF INTEREST

FJR receives institutional and salary support as (a) a coinvestigator and subcontractor with the Peter MacCallum Cancer Center for an investigator‐initiated trial which receives funding support from Regeneron Pharmaceuticals; and (b) a coinvestigator on a translational research project funded by a Regeneron Pharmaceuticals grant. FJR received travel expenses by MGI Australia and New Zealand. All other authors declare no conflict of interest.

## Supporting information


Supplementary data 1

## Data Availability

Most data generated in this study are included in this article. Any additional data are available from the corresponding author upon reasonable request.
