# Supplementary material for: Expression of CD38 on resting peripheral iNKT cells defines an immature subpopulation with distinct functionality in humans
Source: Immunol Cell Biol. 2025 Dec 26;104(2):109–24. doi: 10.1111/imcb.70074 (PMC12872403; doi:10.1111/imcb.70074)
Supplement: Supplementary file 1 — Supplementary data 1 [file IMCB-104-109-s001.docx]

**Supporting information**

**Expression of CD38 on resting peripheral iNKT cells defines an immature subpopulation with distinct functionality in humans**

Christopher Menne^1,2^, Naeimeh Tavakolinia^2^, Louis Perriman^2,3,4^, Wiebke Moskorz^1^, Christine Cosmovici^1^, Andreas Walker^1^, Lara Olejnik^1^, Katharina Raba^5^, Mei R. M. Du^2,6^, Fernando J. Rossello^2,6,7,8^, Igor E. Konstantinov^2,9,10^, Stuart P. Berzins^3,4,11^, Daniel G. Pellicci^2,12,13^, Jörg Timm^1^

*1: Institute of Virology, Heinrich Heine University, Medical Faculty, Düsseldorf, Germany*

*2: Murdoch Children’s Research Institute, The Royal Children’s Hospital, Melbourne, VIC 3052, Australia*

*3: The Fiona Elsey Cancer Research Institute, Ballarat, VIC, Australia.*

*4: Federation University, Ballarat, VIC, Australia.*

*5: Institute for Transplantation Diagnostics and Cell Therapeutics, Medical Faculty, Heinrich-Heine University Düsseldorf, Düsseldorf, Germany*

*6: Novo Nordisk Foundation Center for Stem Cell Medicine, Murdoch Children’s Research Institute, Melbourne, VIC 3052, Australia*

*7: Department of Clinical Pathology, University of Melbourne, Melbourne, VIC, Australia*

*8: Australian Regenerative Medicine Institute, Monash University, Victoria, Australia*

*9: Melbourne Centre for Cardiovascular Genomics and Regenerative Medicine, Melbourne, VIC, Australia.*

*10: Cardiothoracic Surgery, Royal Children's Hospital, Melbourne, VIC, Australia.*

*11: Department of Microbiology and Immunology, Peter Doherty Institute for Infection and Immunity, University of Melbourne, Melbourne, VIC, Australia*

*12: Department of Paediatrics, University of Melbourne, Melbourne, Australia*

*13: Department of Microbiology and Immunology, University of Melbourne, Melbourne, Australia*


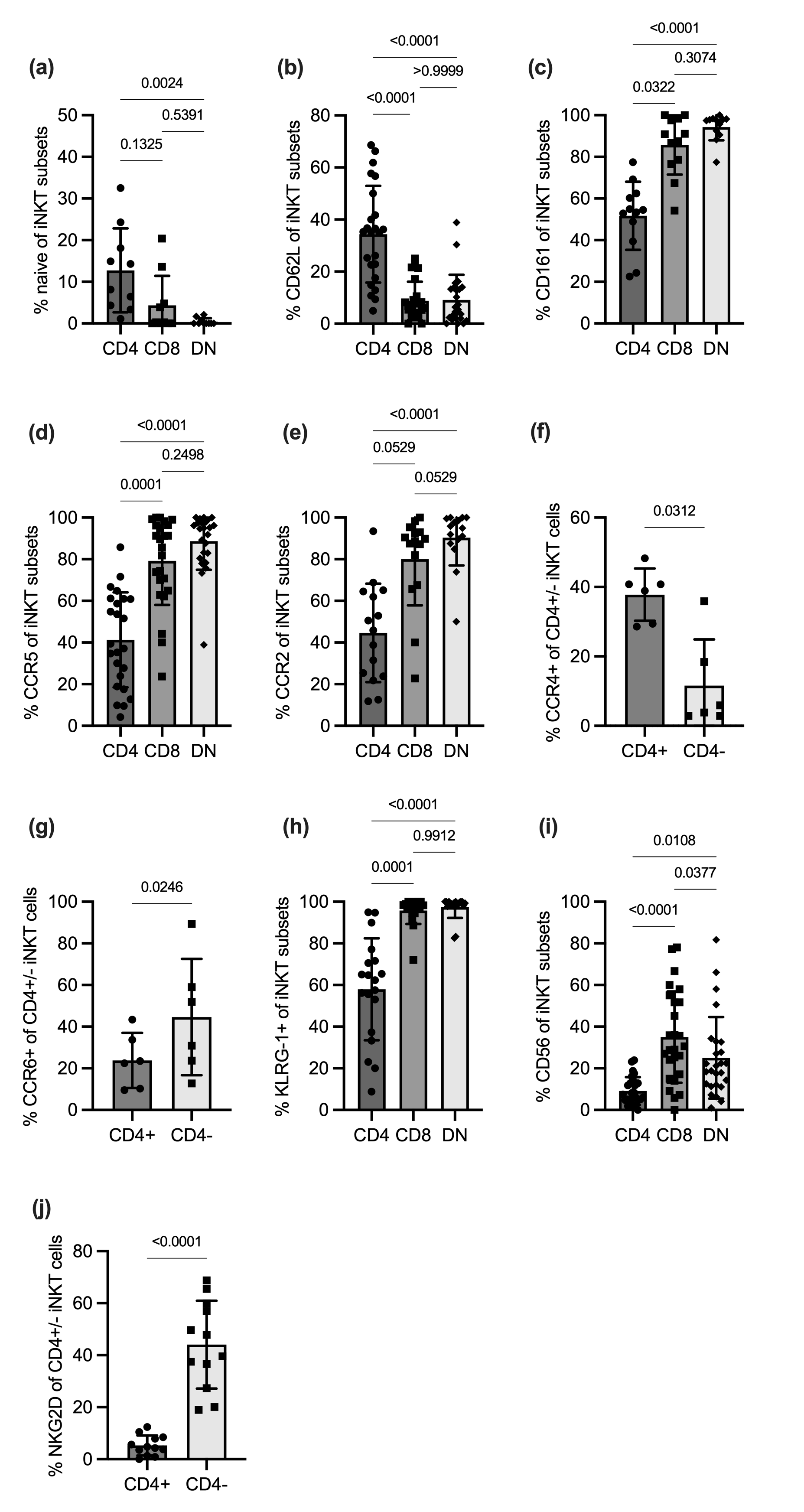


**Supplementary figure 1.** **Frequency of all analysed surface markers on iNKT subsets.** Frequency of **(a)** CD45RA and CCR7 (double positive referred to as naïve) (n = 10), **(b)** CD62L (n = 24), **(c)** CD161 (n = 12), **(d)** CCR5 (n = 24), **(e)** CCR2 (n = 15), **(f)** CCR4 (n = 6), **(g)** CCR6 (n = 6), **(h)** KLRG-1 (n = 19), **(i)** CD56 (n = 26), and **(j)** NKG2D (n = 12) on CD4^+^, CD8^+^ and double negative (DN) iNKT cells was analysed by flow cytometry. Bars represent the mean and error bars show the standard deviation. Samples with 20 or less cells in one of the compared populations were excluded from the analysis. The expression of CCR4 **(f)**, CCR6 **(g)** and NKG2D **(j)** was only subdivided into the subsets of CD4^+^ and CD4^-^ iNKT cells, as the cell numbers were too low to distinguish between CD8^+^ and DN cells. For comparisons between three groups a Friedman test was used **(a – e, h, i)** and a Wilcoxon test **(f)**, or a paired t-test **(g, j)** was performed for comparisons between two groups.


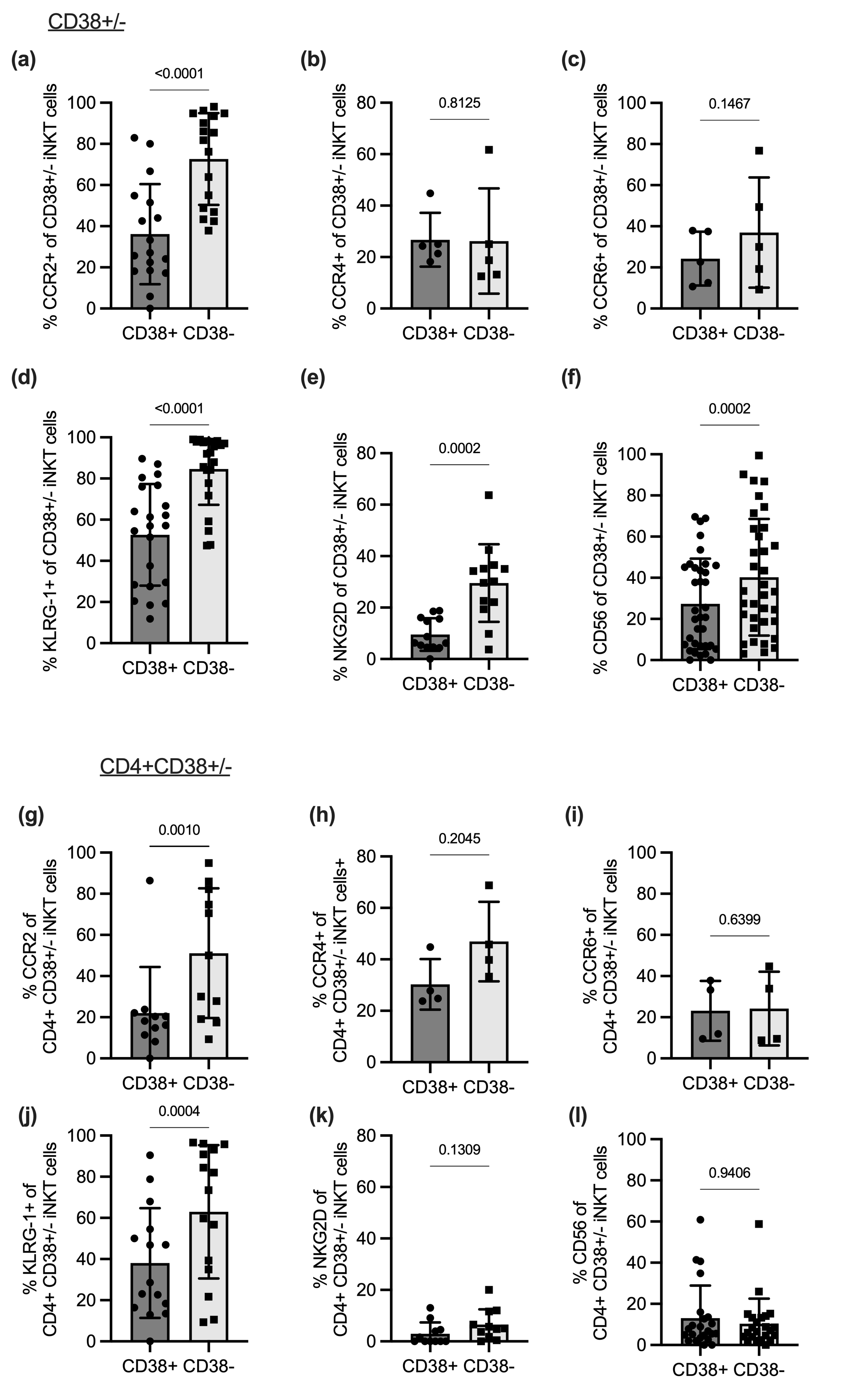


**Supplementary figure 2. Frequency of additional surface markers on CD38^+^ and CD38^-^ iNKT cells.** Frequency of **(a, g)** CCR2 (n = 17 and n = 11), **(b, h)** CCR4 (n = 5 and n = 4), **(c, i)** CCR6 (n = 5 and n = 4), **(d, j)** KLRG-1 (n = 22 and n = 15), **(e, k)** NKG2D (n = 13 and n = 11), and **(f, l)** CD56 (n = 35 and n = 23) on CD38^+^ and CD38^-^ iNKT cells **(a – f)** as well as on CD4^+^ CD38^+^ and CD4^+^ CD38^-^ iNKT cells **(g – l)** was analysed by flow cytometry. Bars represent the mean and error bars show the standard deviation. Donors with 20 or less cells in one of the compared populations were excluded from the analysis. P-values were calculated with a Wilcoxon test **(a, b, d, f, g, j, k, l)** or paired t-test **(c, e, h, i)**.

**
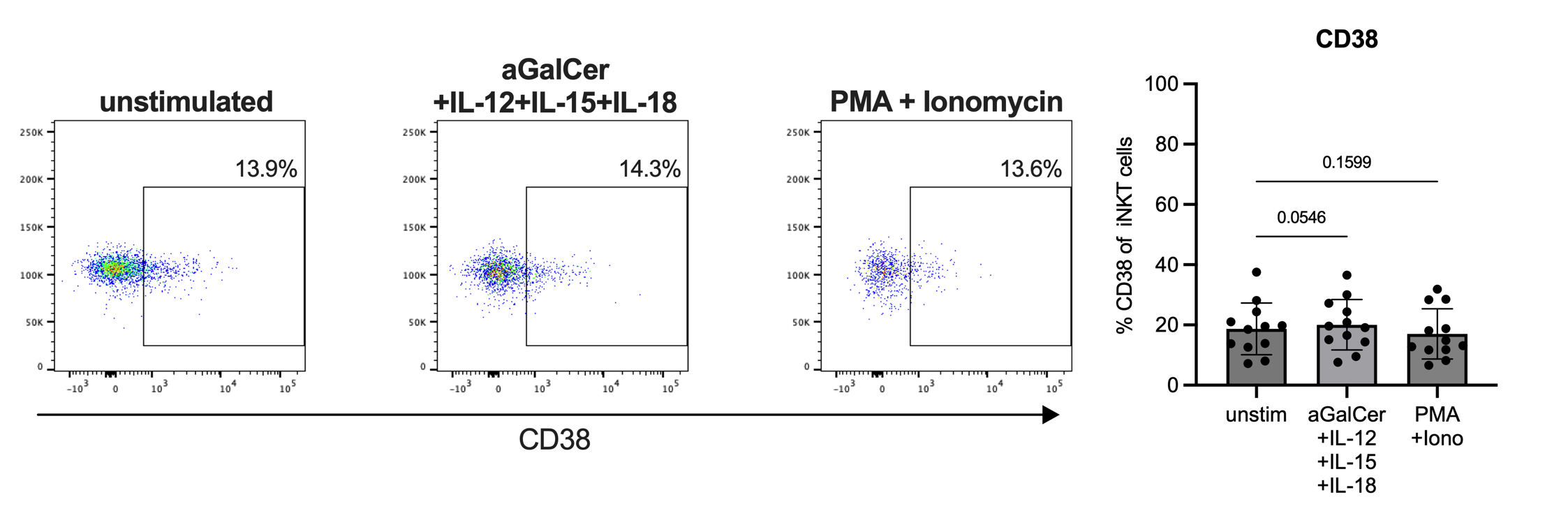
** **Supplementary figure 3. Validation of the in vitro stimulation assay.** CD38 expression on MACS enriched iNKT cells with and without in vitro stimulation with αGalCer and IL-12, IL-15 and IL-18 or PMA and Ionomycin was analysed after eight hours or six hours of stimulation, respectively, in the presence of BFA for the last four hours. A representative FACS plot is shown on the left side and each dot represents one of n = 12 donors in the bar graph on the right side. Groups were compared by one-way ANOVA and the bar depicts the mean of all samples with error bars showing the standard deviation.


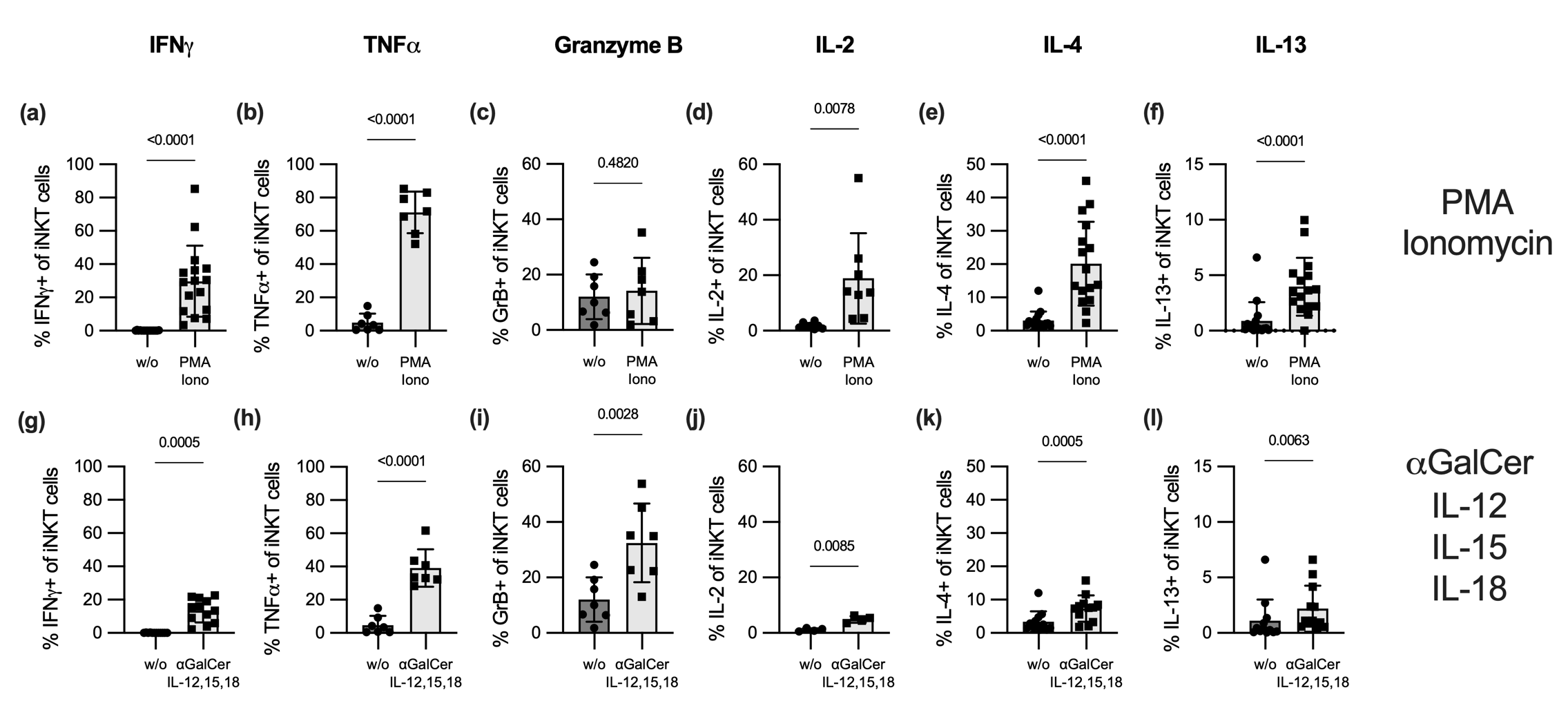
 **Supplementary figure 4. Functional analysis of bulk iNKT cells.** MACS enriched iNKT cells from healthy donor PBMC were stimulated with PMA and Ionomycin for six hours **(a – f)** or αGalCer, IL-12, IL-15 and IL-18 for eight hours **(g – l)** or left unstimulated in the presence of BFA for the last four hours. The production of **(a, g)** IFNγ (n = 16, n = 12), **(b, h)** TNFα (n = 7, n = 7), **(c, i)** Granzyme B (n = 7, n = 7), **(d, j)** IL-2 (n = 8, n = 4), **(e, k)** IL-4 (n = 16, n = 12), and **(f, l)** IL-13 (n = 16, n = 12), was analysed by intracellular cytokine staining. Bars represent the mean, error bars show standard deviation and p-values were calculated with a Wilcoxon test **(a, d, e, f, g, k, l)** or a paired t-test **(b, c, h, i, j)**. Donors with 20 or less cells in one of the compared populations were excluded from the analysis.


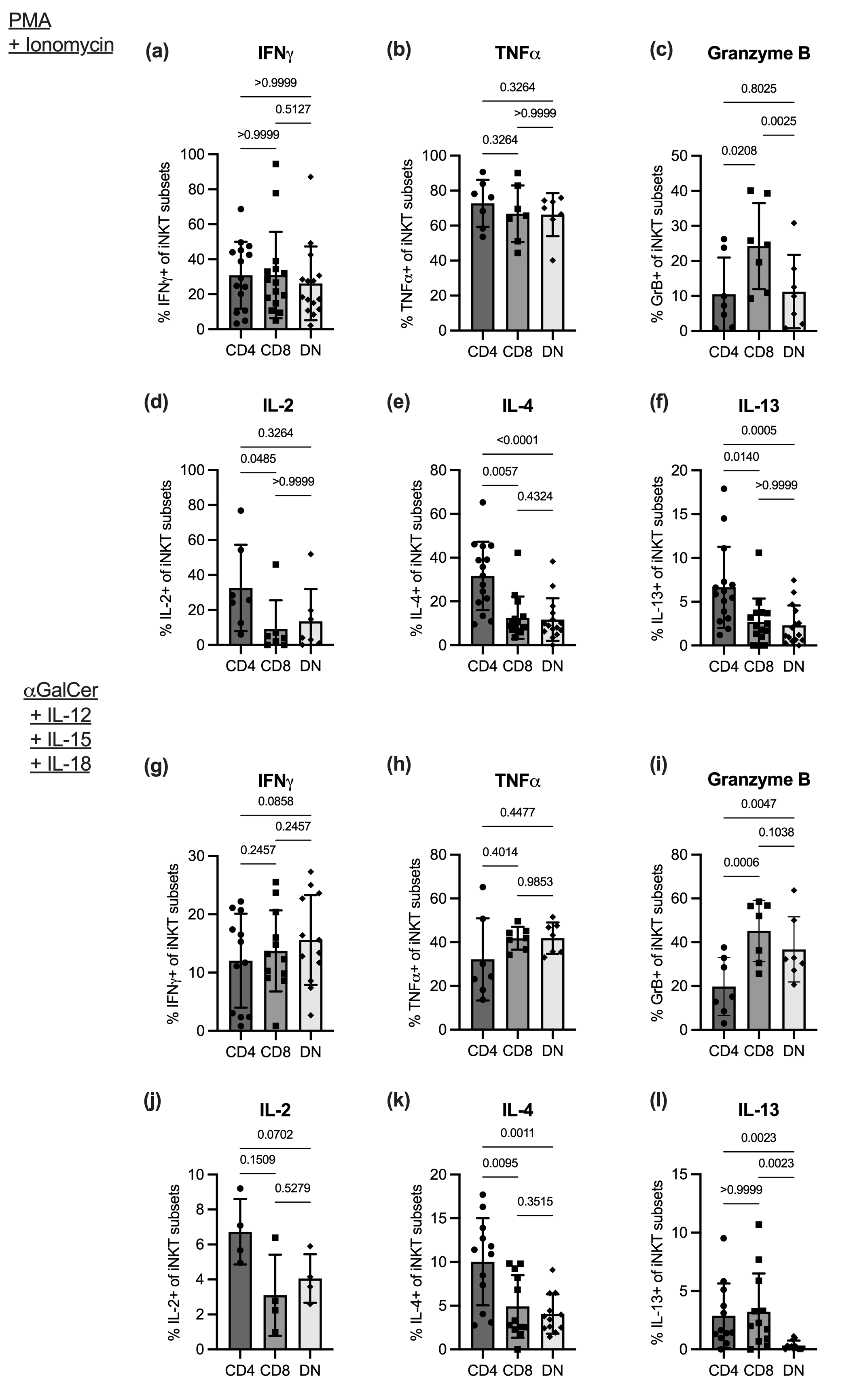


**Supplementary figure 5. Functional analysis of iNKT subsets.** MACS enriched iNKT cells from healthy donor PBMC were stimulated with PMA and Ionomycin for six hours **(a – f)** or with αGalCer and IL-12, IL-15 and IL-18 for eight hours **(g – l)** in the presence of BFA for the last four hours and the production of **(a, g)** IFNγ (n = 15 and n = 12), **(b, h)** TNFα (n = 7 and n = 7), **(c, i)** Granzyme B (n = 7 and n = 7), **(d, j)** IL-2 (n = 7 and n = 4), **(e, k)** IL-4 (n = 15 and n = 12), and **(f, l)** IL-13 (n = 15 and n = 12) by CD4^+^, CD8^+^ and double negative (DN) iNKT cells was analysed by flow cytometry. Bars represent the mean, error bars show standard deviation and groups were compared with a Friedman test **(a, b, d, e, f, l)** or one-way ANOVA **(c, g, h, i, j, k)**. Donors with 20 or less cells in one of the compared populations were excluded from the analysis.


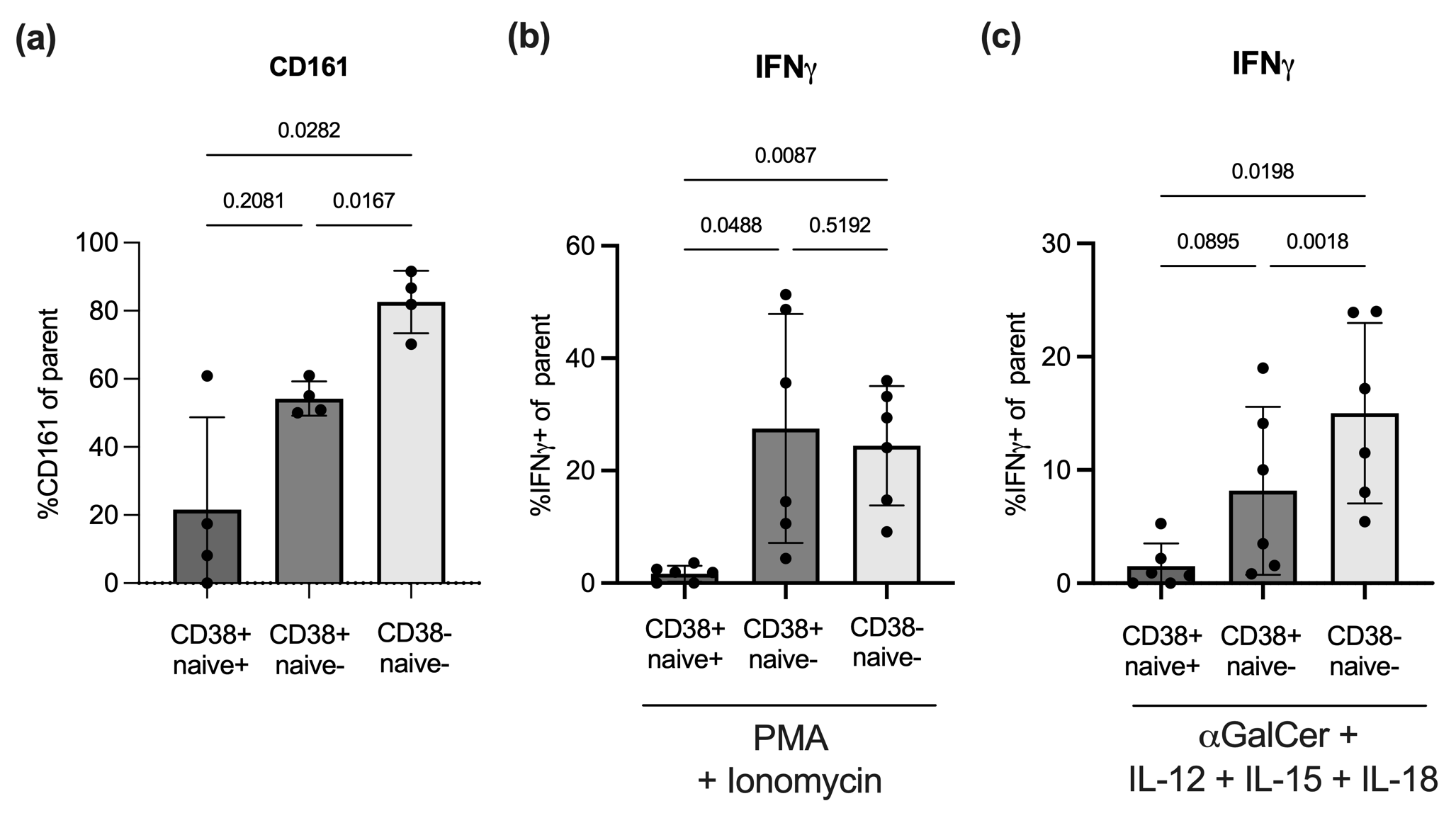


**Supplementary figure 6. Heterogeneity within the CD38+ iNKT cell subset**. **(a)** Frequency of CD161 (n = 4) on CD38+naive+ (CD45RA+CCR7+), CD38+naïve- (non-CCR7+CD45RA+), and CD38- iNKT cells was analysed by flow cytometry. **(b – c)** MACS enriched iNKT cells from healthy donor PBMC were stimulated with PMA and Ionomycin for six hours **(b)** or with αGalCer and IL-12, IL-15 and IL-18 for eight hours **(c)** in the presence of BFA for the last four hours and the production of IFNγ (n = 6) by CD38+naive+ (CD45RA+CCR7+), CD38+naïve- (non-CCR7+CD45RA+), and CD38- iNKT cells was analysed by flow cytometry. Bars represent the mean and error bars show the standard deviation. Samples with 20 or less cells in one of the compared populations were excluded from the analysis. For comparisons between groups a one-way ANOVA was used.


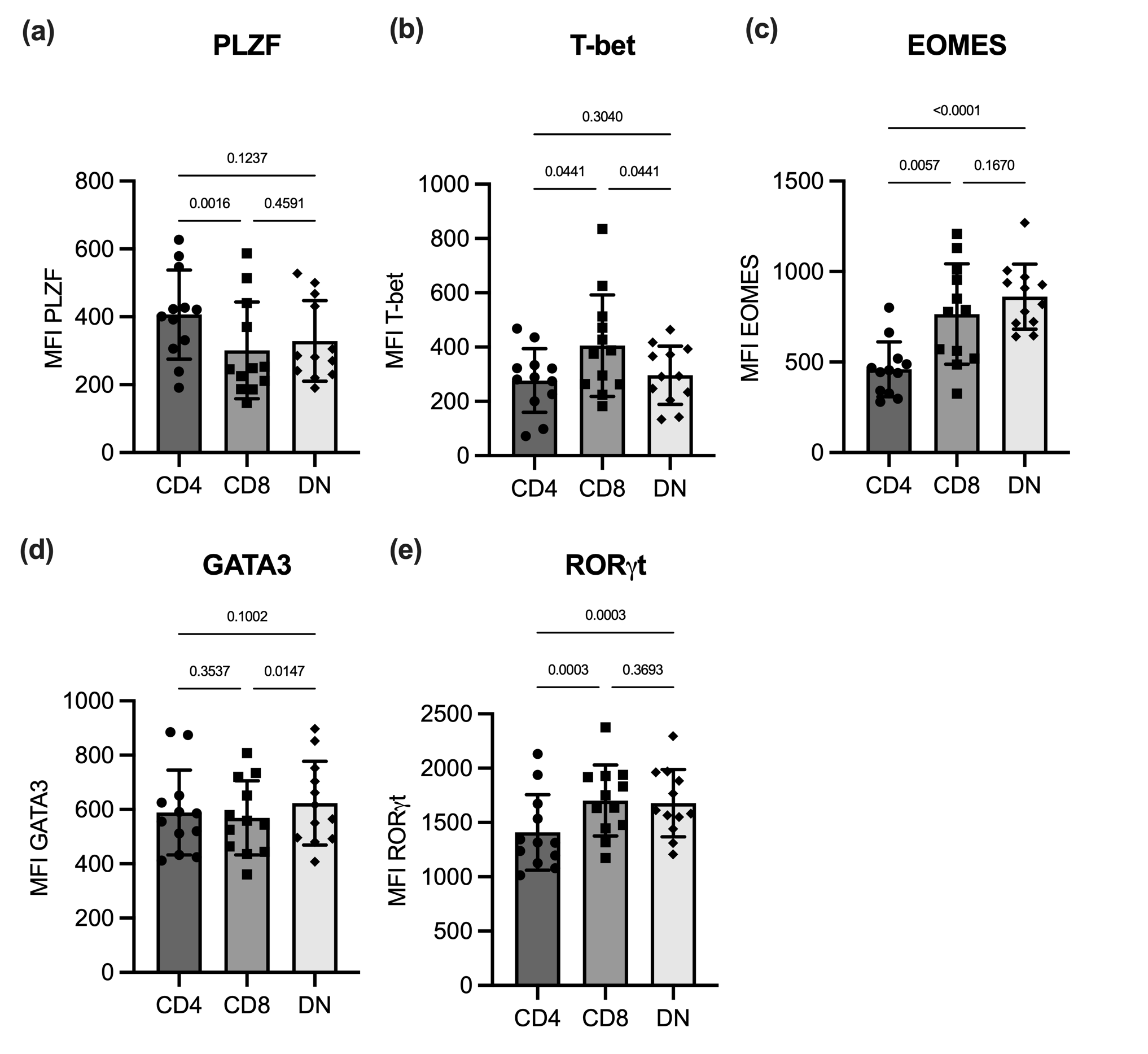


**Supplementary figure 7. Expression of transcription factors by iNKT cell subsets.** Expression (MFI) of **(a)** PLZF, **(b)** T-bet, **(c)** EOMES, **(d)** GATA3, and **(e)** RORγt by CD4^+^, CD8^+^ and double negative (DN) iNKT cells was analysed by flow cytometry. Donors with 20 or less cells in the target gate were excluded from the analysis. Bars represent the mean of n = 12 donors and error bars show the standard deviation. P-values were calculated with a Friedman test **(a)** or one way ANOVA **(b, c, d, e)**.


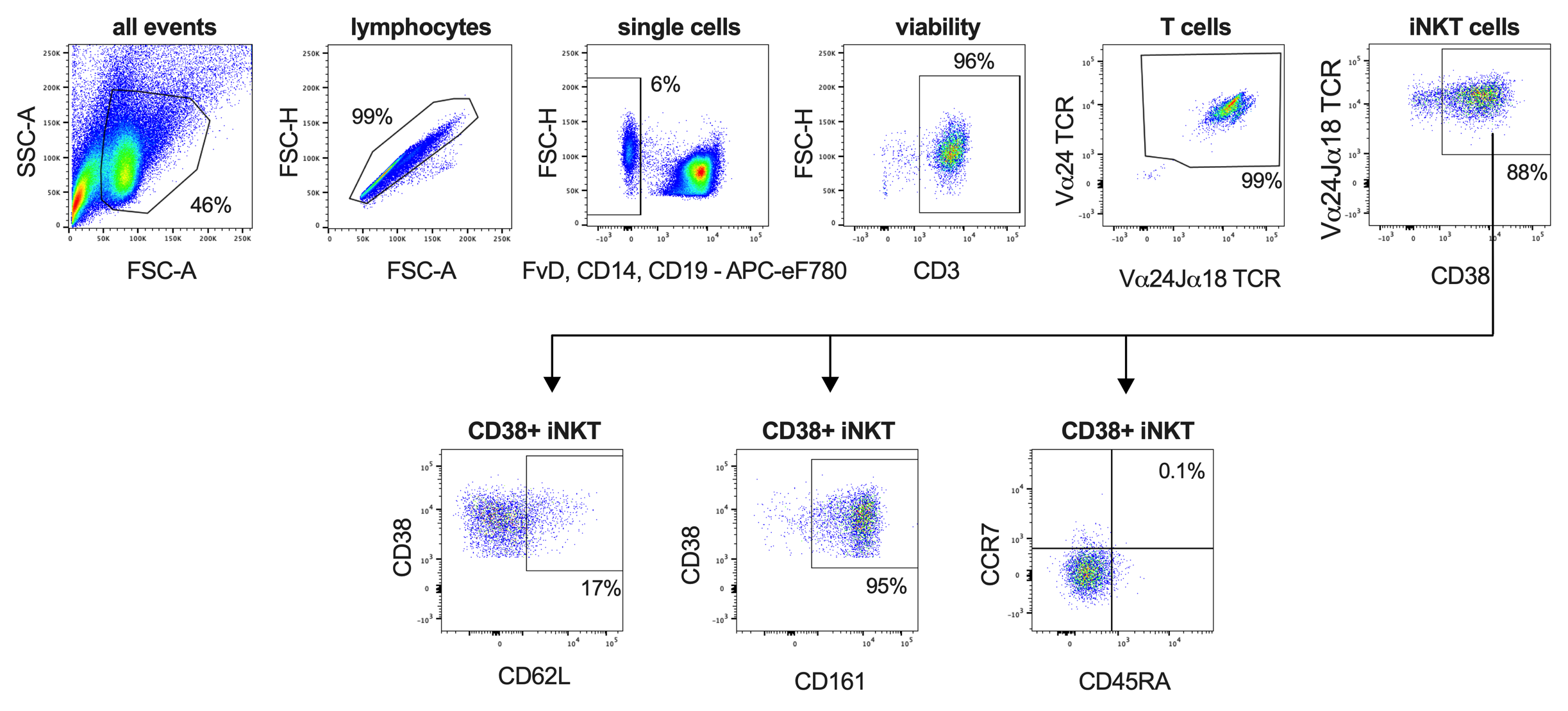


**Supplementary figure 8. Gating strategy of FACS purified iNKT cells after two weeks of in vitro expansion.**


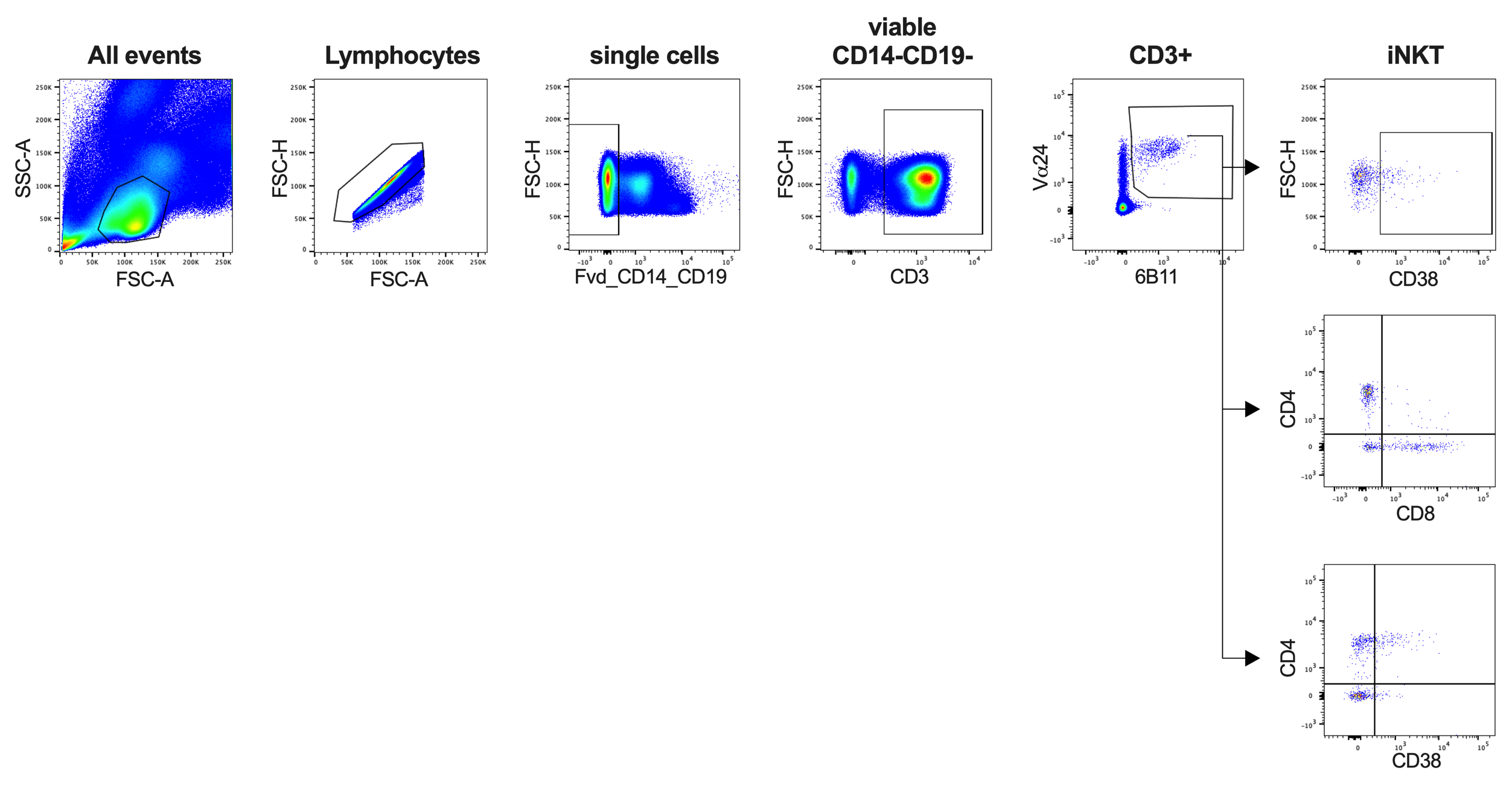


**Supplementary figure 9. Gating strategy of iNKT cells from PBMC.**


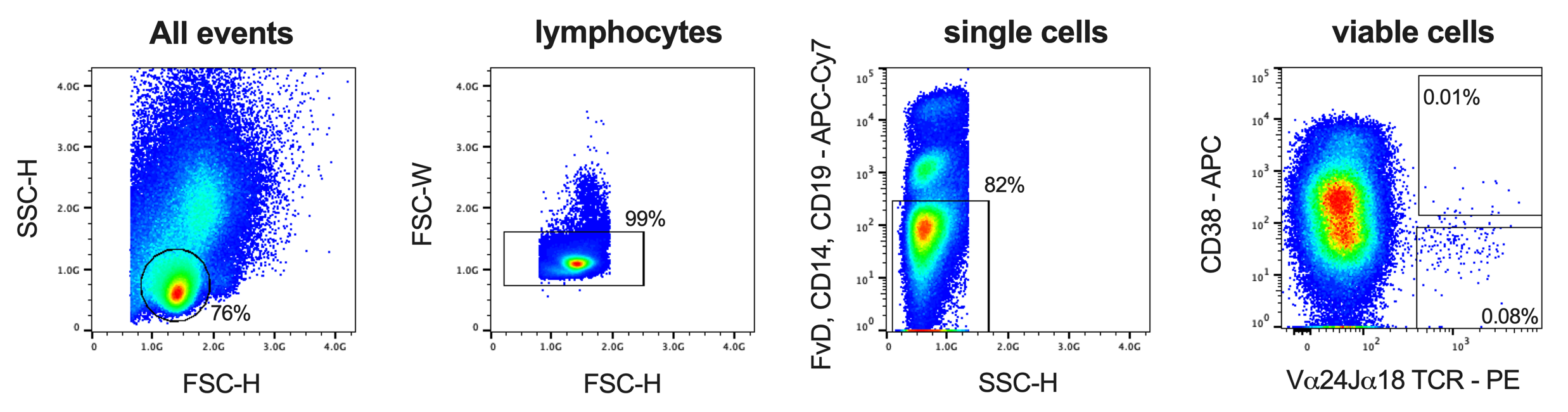


**Supplementary figure 10. FACS gating strategy for CD38+ and CD38- iNKT cells.**

**Supplementary table 1. Staining antibodies and reagents**

| Marker | Fluorochrome | Manufacturer | Cat. no. |
| --- | --- | --- | --- |
| 2B4 | PeCF594 | BD Bioscience, Franklin Lakes, NJ, USA | 564881 |
| 6B11 | BV421 | BD Bioscience, Franklin Lakes, NJ, USA | 342916 |
| 6B11 | PE | Biolegend, San Diego, CA, USA | 342904 |
| 6B11 | APC | Biolegend, San Diego, CA, USA | 342908 |
| 6B11 | PE | BD Bioscience, Franklin Lakes, NJ, USA | 552825 |
| CCR2 | BV421 | Biolegend, San Diego, CA, USA | 357210 |
| CCR4 | APC | Biolegend, San Diego, CA, USA | 359408 |
| CCR5 | BV711 | Biolegend, San Diego, CA, USA | 359130 |
| CCR5 | Pacific Blue | Biolegend, San Diego, CA, USA | 359123 |
| CCR6 | PE/Dazzle594 | Biolegend, San Diego, CA, USA | 353430 |
| CCR7 | PE | Thermo Fisher Scientific, Waltham, MA, USA | 12-1979-42 |
| CCR7 | BV785 | Biolegend, San Diego, CA, USA | 353230 |
| CD127 | BV711 | Thermo Fisher Scientific, Waltham, MA, USA | 407-1278-42 |
| CD14 | APC-eFluor780 | Thermo Fisher Scientific, Waltham, MA, USA | 47-0149-42 |
| CD14 | BV421 | BD Bioscience, Franklin Lakes, NJ, USA | 563743 |
| CD14 | eFluor506 | Thermo Fisher Scientific, Waltham, MA, USA | 69-0149-42 |
| CD14 | BV570 | Biolegend, San Diego, CA, USA | 301831 |
| CD16 | BV785 | Biolegend, San Diego, CA, USA | 302045 |
| CD161 | BV605 | Biolegend, San Diego, CA, USA | 339916 |
| CD161 | PE-Vio770 | Miltenyi, Bergisch Gladbach, Germany | 130-113-594 |
| CD19 | APC-eFluor780 | Thermo Fisher Scientific, Waltham, MA, USA | 47-0199-42 |
| CD19 | eFluor506 | Thermo Fisher Scientific, Waltham, MA, USA | 69-0199-42 |
| CD19 | BV570 | Biolegend, San Diego, CA, USA | 302236 |
| CD27 | BV786 | BD Bioscience, Franklin Lakes, NJ, USA | 563327 |
| CD3 | Alexa Fluor 700 | Biolegend, San Diego, CA, USA | 300324 |
| CD3 | PerCP-Cy5.5 | Thermo Fisher Scientific, Waltham, MA, USA | 45-0037-42 |
| CD3 | APC-H7 | BD Bioscience, Franklin Lakes, NJ, USA | 560176 |
| CD3 | BUV395 | BD Bioscience, Franklin Lakes, NJ, USA | 563546 |
| CD31 | Pe/Dazzle594 | Biolegend, San Diego, CA, USA | 303130 |
| CD38 | PE-Cy7 | Thermo Fisher Scientific, Waltham, MA, USA | 25-0389-42 |
| CD38 | APC | Thermo Fisher Scientific, Waltham, MA, USA | 17-0389-42 |
| CD38 | BV605 | Biolegend, San Diego, CA, USA | 303532 |
| CD38 | BUV496 | BD Bioscience, Franklin Lakes, NJ, USA | 612946 |
| CD39 | BV711 | Biolegend, San Diego, CA, USA | 328228 |
| CD4 | BV786 | BD Bioscience, Franklin Lakes, NJ, USA | 740962 |
| CD4 | Pe-CF594 | BD Bioscience, Franklin Lakes, NJ, USA | 562281 |
| CD4 | BV605 | BD Bioscience, Franklin Lakes, NJ, USA | 562658 |
| CD4 | PE | Thermo Fisher Scientific, Waltham, MA, USA | 12-0045-42 |
| CD4 | BV510 | Biolegend, San Diego, CA, USA | 344634 |
| CD4 | BUV661 | BD Bioscience, Franklin Lakes, NJ, USA | 612962 |
| CD45RA | PE-Cy7 | Thermo Fisher Scientific, Waltham, MA, USA | 25-0458-42 |
| CD45RA | APC | Thermo Fisher Scientific, Waltham, MA, USA | 17-0458-42 |
| CD45RA | PerCP-Cy5.5 | BD Bioscience, Franklin Lakes, NJ, USA | 563429 |
| CD49d | PE | Biolegend, San Diego, CA, USA | 328303 |
| CD56 | PerCP-eFluor710 | Thermo Fisher Scientific, Waltham, MA, USA | 46-0567-42 |
| CD56 | BV650 | Biolegend, San Diego, CA, USA | 362532 |
| CD62L | BV650 | Biolegend, San Diego, CA, USA | 304832 |
| CD62L | PE-Cy7 | Thermo Fisher Scientific, Waltham, MA, USA | 25-0629-42 |
| CD69 | APC | Biolegend, San Diego, CA, USA | 310910 |
| CD73 | PeCF594 | Biolegend, San Diego, CA, USA | 344020 |
| CD8 | BV510 | Biolegend, San Diego, CA, USA | 301048 |
| CD8 | APC | BD Bioscience, Franklin Lakes, NJ, USA | 564791 |
| CD8 | FITC | Thermo Fisher Scientific, Waltham, MA, USA | 11-0088-42 |
| CD8 | BV650 | Biolegend, San Diego, CA, USA | 301042 |
| CD8 | BUV805 | BD Bioscience, Franklin Lakes, NJ, USA | 564912 |
| CD8 | PeCF594 | BD Bioscience, Franklin Lakes, NJ, USA | 562282 |
| CD8 | BUV805 | BD Bioscience, Franklin Lakes, NJ, USA | 564912 |
| CD8 | PeCF594 | BD Bioscience, Franklin Lakes, NJ, USA | 562282 |
| CD8 | AF700 | Biolegend, San Diego, CA, USA | 301028 |
| CRTH2 | FITC | Biolegend, San Diego, CA, USA | 350108 |
| EOMES | PerCP-Cy5.5 | Thermo Fisher Scientific, Waltham, MA, USA | 46-4877-41 |
| FasL | BV605 | BD Bioscience, Franklin Lakes, NJ, USA | 744099 |
| Fixable Viability Dye (FvD) | eF780 | Thermo Fisher Scientific, Waltham, MA, USA | 65-0865-18 |
| Fixable Viability Dye (FvD) | eF506 | Thermo Fisher Scientific, Waltham, MA, USA | 65-0866-18 |
| GATA3 | PE-CF594 | BD Bioscience, Franklin Lakes, NJ, USA | 563510 |
| Granzyme B | PE/Dazzle594 | Biolegend, San Diego, CA, USA | 372216 |
| Granzyme B | PeCy7 | Biolegend, San Diego, CA, USA | 372214 |
| Granzyme B | APC-Fire750 | Biolegend, San Diego, CA, USA | 372210 |
| IFNg | BV421 | BD Bioscience, Franklin Lakes, NJ, USA | 564791 |
| IFNg | BV711 | Biolegend, San Diego, CA, USA | 502540 |
| IL-10 | BV421 | Biolegend, San Diego, CA, USA | 501421 |
| IL-13 | VioR667 | Miltenyi, Bergisch Gladbach, Germany | 130-120-178 |
| IL-2 | PerCP-eFluor710 | Thermo Fisher Scientific, Waltham, MA, USA | 46-7029-42 |
| IL-4 | PE-CF594 | Biolegend, San Diego, CA, USA | 500832 |
| KLRG-1 | PE | Miltenyi, Bergisch Gladbach, Germany | 130-120-426 |
| NKG2D | PerCP-Cy5.5 | Thermo Fisher Scientific, Waltham, MA, USA | 46-5878-42 |
| PD-1 | BV421 | Biolegend, San Diego, CA, USA | 329920 |
| PD-1 | BV650 | BD Bioscience, Franklin Lakes, NJ, USA | 564104 |
| PLZF | Alexa Fluor 647 | BD Bioscience, Franklin Lakes, NJ, USA | 563490 |
| RORgt | PE | Miltenyi, Bergisch Gladbach, Germany | 130-123-248 |
| T-bet | BV711 | Biolegend, San Diego, CA, USA | 644820 |
| TNFa | APC | Thermo Fisher Scientific, Waltham, MA, USA | 17-7349-82 |
| TNFa | BV785 | Biolegend, San Diego, CA, USA | 502948 |
| TNFa | PeCy7 | Biolegend, San Diego, CA, USA | 502930 |
| Va24 | FITC | Beckman Coulter, Brea, CA, USA | IM1589 |
| Va24 | PE | Miltenyi, Bergisch Gladbach, Germany | 130-115-786 |
| Va24 | FITC | Miltenyi, Bergisch Gladbach, Germany | 130-115-785 |
| Zombie | NIR | Biolegend, San Diego, CA, USA | 423106 |
| CellTrace™ Violet Cell Proliferation Kit |  | Thermo Fisher Scientific, Waltham, MA, USA | C34557 |

**Supplementary table 2. Flow cytometry panels**
